# High atomic weight, high-energy radiation (HZE) induces transcriptional responses shared with conventional stresses in addition to a core “DSB” response specific to clastogenic treatments

**DOI:** 10.3389/fpls.2014.00364

**Published:** 2014-08-01

**Authors:** Victor Missirian, Phillip A. Conklin, Kevin M. Culligan, Neil D. Huefner, Anne B. Britt

**Affiliations:** ^1^Department of Plant Biology, University of California DavisDavis, CA, USA; ^2^Department of Molecular, Cellular, and Biomedical Sciences, University of New HampshireDurham, NH, USA

**Keywords:** DNA repair, double-strand breaks, transcriptomics, stress, cell-cycle, ionizing radiation, HZE, gamma radiation

## Abstract

Plants exhibit a robust transcriptional response to gamma radiation which includes the induction of transcripts required for homologous recombination and the suppression of transcripts that promote cell cycle progression. Various DNA damaging agents induce different spectra of DNA damage as well as “collateral” damage to other cellular components and therefore are not expected to provoke identical responses by the cell. Here we study the effects of two different types of ionizing radiation (IR) treatment, HZE (1 GeV Fe^26+^ high mass, high charge, and high energy relativistic particles) and gamma photons, on the transcriptome of *Arabidopsis thaliana* seedlings. Both types of IR induce small clusters of radicals that can result in the formation of double strand breaks (DSBs), but HZE also produces linear arrays of extremely clustered damage. We performed these experiments across a range of time points (1.5–24 h after irradiation) in both wild-type plants and in mutants defective in the DSB-sensing protein kinase ATM. The two types of IR exhibit a shared double strand break-repair-related damage response, although they differ slightly in the timing, degree, and ATM-dependence of the response. The ATM-dependent, DNA metabolism-related transcripts of the “DSB response” were also induced by other DNA damaging agents, but were not induced by conventional stresses. Both Gamma and HZE irradiation induced, at 24 h post-irradiation, ATM-dependent transcripts associated with a variety of conventional stresses; these were overrepresented for pathogen response, rather than DNA metabolism. In contrast, only HZE-irradiated plants, at 1.5 h after irradiation, exhibited an additional and very extensive transcriptional response, shared with plants experiencing “extended night.” This response was not apparent in gamma-irradiated plants.

## Introduction

Programmed responses to DNA damage include the induction of repair, recombination, mutagenesis, cell cycle arrest, and cell death. These responses vary with the quality and quantity of the damage induced, with the phase of the cell cycle (Jazayeri et al., 2006), and with cell type (Shi et al., 1997). Damage response can also be influenced by environmental inputs (Shor et al., 2013), the age of the organism (Goukassian et al., 2000; Gredilla et al., 2012; Garm et al., 2013), and even the time of day (Ramsey and Ellisen, 2011; Gaddameedhi et al., 2012). A thorough knowledge of damage response provides insight into the mechanisms that promote genetic stability. In addition, comparative studies of damage response (the study of response to different agents, in different environments, or in different cell types) inform us as to how organisms balance the benefits of error-free repair vs. the risks engendered by cell death, growth arrest, inappropriate repair and ectopic recombination.

DNA damage response (DDR) is highly complex, involving the regulation of gene expression at all mechanistic levels and affecting the expression of thousands of genes. For this reason, DDR is an excellent subject for proteomic and transcriptomics approaches. Studies (largely focused on Arabidopsis seedlings) of both the transcriptomic and the phenotypic consequences of a variety of DNA damaging agents (Chen et al., 2003; Ulm and Nagy, 2005; Culligan et al., 2006; Kim, 2006; Ricaud et al., 2007; Cools et al., 2011; Mannuss et al., 2012) have led to the conclusion that different DNA damaging agents induce very different phenotypic and transcriptomic responses. This is worth considering in depth, as all significant types of DNA damage might be naively expected to have very similar immediate physiological consequences, for example, the blockage of transcription and replication. Observed differences in response to DNA damaging agents may not be due to response to DNA damage *per se*. All “DNA damaging agents” also damage other cellular components, and some may act as signals (i.e., UV-B, ROS) that invoke responses unrelated to DNA repair. The ability of a species or cell to cope -or not- with this “collateral damage” can in some instances determine the difference between life and death- as exemplified by the role of protein-protective compounds in conferring extreme radioresistance in some bacteria (Daly et al., 2007).

In this study we focus on the quantitative, qualitative, and temporal differences in transcriptional response to two different types of ionizing radiation (IR): gamma photons, which have low rates of linear energy transfer (LET), and relativistic Fe nuclei (here termed HZE), a high LET form of IR. Interest in the differences in biological response to these two forms of IR has risen as long-term manned missions beyond the shielding effects the Earth's atmosphere and magnetic field- to the moon and to Mars- have been increasingly contemplated.

Gamma photons interact weakly with matter- a large fraction of gamma photons will pass though a cell's nucleus without losing any of their energy at all. However, some fraction of photons, during their transit across the cell, will interact indiscriminately with the cell's molecules via Compton scattering. In this process, a small fraction of the photon's energy will be transferred to an atom, inducing the ejection of a high-energy electron. The ejected electron will proceed to lose its energy through interactions with many additional atoms, producing many additional radicals. This energy will be lost quickly (on the order of 10^−6^ s), resulting in “clusters” of ionization events. Typically, 2–5 radicals are induced per cluster along a track length of 4–5 nm- on the order of the diameter of DNA (2 nm). It is the clustered nature of the formation of radicals that distinguishes the damage induced by IR from the damage induced by radical-forming chemicals (such as hydrogen peroxide or heavy metals), which produce isolated radicals (nicely reviewed in Ward, 1998). Isolated radicals, on interaction with DNA, induce singly damaged sites, which can be corrected via excision repair, using the undamaged strand as a template for error-free repair. In contrast, excision repair is not an option for DNA that has suffered the formation of closely spaced damage on both strands of the DNA. Thus, although all types of molecules in the cell are damaged by IR, in eukaryotic cells the biological effects of IR (mutation, cell cycle arrest, and cell death) are ascribed to the induction of clustered lesions in DNA.

Relativistic (near light-speed) Fe^26+^ nuclei also induce high-energy electrons and so produce these scattered “handfuls” of clustered radicals. However, *in addition*, as this pinpoint charge source travels through the cell, it *continuously* displaces high-energy electrons from molecules along its path. Thus, this high LET particle (170 keV/μ, in contrast to gamma radiation's average of 0.2 keV/μ) lays down a very dense and continuous cylinder of radicals as it crosses the cell. DNA molecules in the direct path of these particles are inevitably damaged at multiple sites. This produces a pattern of co-located multiply damaged sites– a continuous linear array of clusters on neighboring DNA molecules, facilitating the formation of deletions, inversions, and translocations. Isolated clusters and singly damaged sites occur also, at molecules in the less dense “penumbra” of secondary electrons generated at the periphery of the particle's path (Magee and Chatterjee, 1980).

IR interacts indiscriminately with all molecules, and so damages all molecules. Researchers focus on DNA damage because DNA is uniquely low copy number and hence irreplaceable, and because the radiosensitivity of DNA repair and response mutants clearly indicates that DNA repair plays a central role in alleviating the mutagenic, carcinogenic, and toxic effects of IR. However, the very dense and collocated track of radicals laid down by HZE, and the inevitable damage to proteins, protein complexes, and membranes may have significant physiological effects that have not yet been characterized.

The effects of gamma radiation on plant growth, development, and mutation have, in contrast, been extensively described in several plant species. Most recently attention has focused on the Arabidopsis embryo and seedling, where gamma radiation has been shown to induce programmed cell death, cell cycle arrest, premature differentiation, and mutation (Preuss and Britt, 2003; Hefner et al., 2006; Fulcher and Sablowski, 2009; Furukawa et al., 2010). These effects are enhanced in mutants defective in DSB repair (via NHEJ pathways) (West et al., 2000; Tamura et al., 2002; Friesner and Britt, 2003; Hefner et al., 2003; Heacock et al., 2007; Fulcher and Sablowski, 2009), suggesting that clustered lesions are responsible for most of the effects of gamma radiation. Gamma radiation has also been shown (again, in the Arabidopsis seedling) to induce a robust transcriptional response, in which DNA and RNA metabolism genes are over-represented (Culligan et al., 2006; Ricaud et al., 2007). These same repair genes—most involved in HR- are also induced by treatment with bleomycin (BLM, an agent that induces clustered damage) plus mitomycin C (MMC, an inter-strand crosslinking agent) (Chen et al., 2003; Roa et al., 2009). Induction of these repair-related transcripts requires the DSB-sensing protein kinase ATM, suggesting that this is a direct response to the induction of DSBs. The transcriptional effects of HZE, in contrast, have not been described in any plant species.

Here we compare the time course (from 1.5 to 24 h after irradiation) of the transcriptional response to gamma radiation vs. HZE. We find that both agents strongly induce, a set of double-strand-break-repair-related transcripts, although the intensity and the degree of ATM-dependence of the response differs with the two types of radiation. We also describe the induction of additional transcripts, without known roles in DNA repair, induced specifically by HZE, which may reflect a response to damage to other (non-chromosomal) cellular components. Lastly, we contrast the transcriptional response to both types of IR to previously published data sets describing responses to a wide variety of more conventional stresses.

## Materials and methods

### Growth and irradiation of seedlings

Eight days prior to irradiation, wild-type Ws and *atm-1* seeds were surface sterilized using a 20% bleach solution and then plated on 1 × MS, Phytoagar (PlantMedia, BioWorld, Dublin, OH). These plates were placed on ice and shipped to Brookhaven National Labs (BNL) where they were placed at 4°C. Five days prior to irradiation, the plates were placed vertically in a 21°C growth chamber in the BNL Controlled Environment Facility, where plants grew under cool white lamps (16 h day) until the time of irradiation. For irradiation, plates were moved either to the Controlled Environment Radiation Facility for exposure to gamma radiation (100 Gy at 7 Gy/min), or to the National Space Radiation Laboratory (NSRL) for exposure to accelerated 1 GeV/n ^56^Fe particles (30 Gy at 7 Gy/min). NSRL is located on the BNL campus. Plants exposed to ^56^Fe particles were placed in a lighted hood for approximately 30 min, at which time the samples were considered to be deactivated and safe for return to the growth chamber, where they remained until time of harvest. Both facilities are located on the BNL campus.

### Preparation of tissue for microarray analysis

Whole wt seedlings were harvested 1.5, 3, 6, 12, and 24 h after treatment, frozen in liquid Nitrogen, and stored at −80°C. *atm-1* seedlings were harvested in parallel, though seedlings were not collected at 3 h. Isolated total RNA samples were processed as recommended by the manufacturer (Affymetrix GeneChip Expression Analysis Technical Manual, Affymetrix Inc.). Approximately 30–40 plants were harvested and pooled together for total RNA extraction (RNeasy Mini-Prep; Qiagen, Valencia, CA, USA). Eluted total RNAs were quantified with a portion of the recovered total RNA adjusted to a final concentration of 1.25 μg μl^−1^.

RNA quality control, cRNA production and hybridization were performed at U.C. Irvine's Microarray Core Facility using the following protocol: All starting total RNA samples were quality assessed prior to beginning target preparation/processing steps by running out a small amount of each sample (typically 25–250 ng well^−1^) onto a RNA Lab-On-A-Chip (Caliper Technologies Corp., Mountain View, CA, USA) that was evaluated on an Agilent Bioanalyzer 2100 (Agilent Technologies, Palo Alto, CA, USA). Single-stranded and then double-stranded cDNA was synthesized from the poly(A)+ mRNA present in the isolated total RNA (typically 10 μg total RNA starting material for each sample reaction) using the SuperScript double-stranded cDNA synthesis kit (Invitrogen, Carlsbad, CA, USA) and poly(T) nucleotide primers that contained a sequence recognized by T7 RNA polymerase. A portion of the resulting double-stranded cDNA was used as a template to generate biotin-tagged cRNA from the Affymetrix GeneChip IVT labeling kit, and 15 μg of the resulting biotin-tagged cRNA was fragmented to an average strand length of 100 bases (range 35–200 bases) following prescribed protocols (Affymetrix GeneChip Expression Analysis Technical Manual). Subsequently, 10 μg of this fragmented target cRNA was hybridized at 45°C with rotation for 16 h (Affymetrix GeneChip Hybridization Oven 640) to probe sets present on an Affymetrix ATH1 array. The GeneChip arrays were washed and then stained with SAPE (streptavidin–phycoerythrin) on an Affymetrix Fluidics Station 450, followed by scanning on a GeneChip Scanner 3000. The results were quantified and analyzed using GCOS 1.2 software (Affymetrix Inc.) with default values (scaling, target signal intensity = 500; normalization, all probe sets; parameters, all set at default values).

### Differential expression analysis of microarray data

Normalization of microarray data was performed using the R (Team, 2012) package RMA (Irizarry et al., 2003) for ATH1 arrays (including those from our own experiments) and the package BufferedMatrixMethods (Benjamin Milo Bolstad. BufferedMatrix: A matrix data storage object held in temporary files. R package version 1.22.0. <http://www.bmbolstad.com>) for 1.0R tiling arrays (none of which were from our own experiments). Any 1.0F arrays were excluded from analysis. Sets of differentially expressed transcripts were determined using the R package Limma (Smyth, 2005), applying a significance threshold of adjusted *p*-value < 0.05 [*p*-values of differential expression were adjusted using the Benjamini–Hochberg multiple testing method, which should control the expected false discovery rate (Benjamini and Hochberg, 1995)].

### Elimination of transcripts responsive to circadian and developmental effects

Irradiated wild-type seedlings were collected at 1.5, 3, 6, 12, and 24 h after irradiation. Irradiated *atm-1* seedlings were collected at 1.5, 6, 12, and 24 h after irradiation. Unirradiated controls were collected only at 1.5 and 24 h. For this reason, in the majority of our figures, fold-induction by IR is illustrated, effects discussed, and conclusions drawn solely based on the data from the 1.5 to 24 h time points. In addition, transcripts with fold induction (or suppression) of 2-fold or less are not considered in this manuscript, though the interested reader can access that data online (XXXcite source site). In some Supplementary Figures (Figures S2, S4, S5, S6), the intermediary time points are included in order to allow the reader to roughly determine the duration and peak of the effects. In order to avoid possible confounding effects of circadian influences on these intermediary data sets, transcripts known to be subject to strong circadian regulation [2-fold or more (Covington and Harmer, 2007), 586 transcripts] have been deleted from consideration throughout this paper, as have transcripts that differ 2-fold or more between the unirradiated 1.5 and 24 h controls (685 transcripts). These two deleted gene sets share 85 transcripts. These gene sets are listed in Table S1, and, again, the reader can access the unredacted data set online.

As an additional check for possible diurnal effects stemming from the absence of a control for each intermediary time point, we extended the expression profiles in selected figures to include the circadian time series expression profiles on which our circadian filtering method was based (Covington and Harmer, 2007) as well as time series expression profiles for the diurnal regulation of 7-to-9-day-old Landsberg erecta seedlings under a light/dark cycle (16 h day, 8 h night) that matched our own growth conditions (Michael et al., 2008). Circadian and diurnal profiles were scaled- separately, and for each transcript- so that the minimum (or maximum, depending on the figure) fold change would be zero.

### Hierarchical clustering and visualization of expression profiles (dendrograms)

Hierarchical clustering of transcripts with a fold change >2 and an adjusted *p*-value < 0.05 in at least one of the 1.5 or 24-h timepoints/treatments was performed with the use of the program Cluster 3.0. Average-linkage clustering of genes was calculated with a correlation cutoff of 0.8 and an exponent of 1.0. The resulting clusters were visualized with the use of the program TreeView as previously described (Eisen et al., 1998). Cluster 3.0 and Treeview software is available at http://rana.lbl.gov/EisenSoftware.htm.

### Semi-quantitative PCR

To determine the dose response for key DSB repair transcripts *BRCA1* and *RAD51*, semiquantitative RT-PCR was performed on cDNA isolated from 5 day old seedlings 1.5 h after completion 100 Gy gamma radiation at a dose rate of 1.8 Gy/min. The seedlings were frozen in liquid N_2_ and RNA was immediately isolated with Trizol reagent (Invitrogen). Two micrograms of the total RNA was reverse transcribed with Superscript III (Invitrogen) according to the manufacturer's protocol. Semi-quantitative PCR was conducted with the Bio-Rad C1000 thermocycler using 22 cycles. The primers used for BRCA1 and Rad51 are:

BRCA1F2 (GGATGGGAAGAGAACTCAAGTGC),
BRCA1rtR2 (GTTGCTCGTCTTCCTTCGATGG),
Rad51AF1 (GGTGTTGCTTATACTCCGAGGAAGG), and
Rad51ArtR1 (CAGCCACACCAAACTCATCTGCTAAC).
*Elf4A* was used as a loading control with primers
elF4A-(CTCTCGCAATCTTCGCTCTTCTCTTT) and
elf4A-5 (TCATAGATCTGGTCCTTGAAAC).

### External sources of microarray data

Our expression data on HZE and Gamma radiation was compared against existing microarray experiments for the treatment of Arabidopsis with a variety of individual abiotic or biotic stresses. We included data on Cold, Heat, Drought, Salt, Osmotic, Genotoxic (Bleomycin, a DSB-inducing agent, plus Mitomycin C, a crosslinking agent), UV-B, and Wounding, from the AtGenExpress abiotic stress data set (Kilian et al., 2007) In addition, we compared against data for stress treatments with Paraquat (De Coninck et al., 2013), *Pseudomonas syringae* (with and without the effector AvrRpt2) (Zheng et al., 2012), Hydroxyurea (Cools et al., 2011), and Extended Night (the extension of the length of night- via a delayed dawn, rather than an early sunset) (Usadel et al., 2008).

### Gene ontology enrichment analysis

Gene Ontology (Ashburner et al., 2000) enrichment analysis was performed using DAVID (Huang da et al., 2009a,b).

### Heat-maps displaying overlaps between sets of “top 100” induced transcripts

Top 100 gene sets were computed by ranking the set of all significantly up-regulated (or down-regulated) transcripts by fold change. Significance was determined by an adjusted *p*-value cutoff of 0.05. In addition, transcripts that did not have at least one dedicated ATH1 probe set, that is, a probe set that is uniquely associated with that transcript, were excluded from consideration. For Paraquat, the one stress whose transcriptional response was measured using a tiling 1.0R array, we also excluded transcripts that were not represented in the tiling 1.0R microarray. Since a small percentage of transcripts that have a dedicated ATH1 probe set are not present in the tiling 1.0R array, there is a slight amount of additional bias in computing “top 100” overlaps between the other stresses and Paraquat (relative to the “top 100” overlaps among the other stresses themselves). If the filtering process described above resulted in less than 100 transcripts for a given experimental condition, then that experimental condition was not included in the heat-map in question.

## Results

### Results section 1: overview

In order to compare the effects of gamma radiation to those of HZE, we exposed wild-type (ecotype Ws) 5-day-old seedlings to doses of these agents that were nearly biologically equivalent in their short-term effects on root growth. These doses (100 Gy gamma radiation, 30 Gy 1GeV/nucleon Fe^26+^) were sufficient to induce a transient arrest of root growth, and had some long-term effects on development, fertility, or genetic stability (see accompanying paper). For those familiar with the effects of IR, these doses may appear to be extremely large, and Arabidopsis, therefore, would appear to be extremely radio-resistant. However, the Arabidopsis genome is small (135 Mb) in comparison with the human genome (3.2 Gb). Thus, a 100 Gy gamma radiation dose in Arabidopsis would generate the same number of DSBs as a 4 Gy dose in humans (where the LD_50_ is 5 Gy) (Mole, 1984).

ATM is a protein kinase required in eukaryotes for recognition and signaling of DSBs (Shiloh and Ziv, 2013). Earlier work with gamma radiation has established that ATM regulates many, but not all, aspects of gamma radiation response in Arabidopsis (Garcia et al., 2003; Friesner et al., 2005; Vespa et al., 2005, 2007; Culligan et al., 2006; Jazayeri et al., 2006; Fulcher and Sablowski, 2009; Yoshiyama et al., 2009; Adachi et al., 2011; Amiard et al., 2011). In order to specifically identify HZE-induced responses that are regulated by ATM, we also performed this analysis in the T-DNA insertion mutant *atm-1*. For the mutant, time points were taken at 1.5, 6, 12, and 24 h after irradiation. Unirradiated controls for both of these genotypes were collected at 1.5 and 24 h after irradiation.

“Fold induction or repression,” as described in this paper, refers to relative levels of transcripts of the 1.5, 3, 6, and 12 h irradiated vs. the unirradiated control (collected at the 1.5 h time point). The 24 h point was compared to unirradiated controls collected at 24 h after irradiation. Because of our lack of controls for circadian variation in gene expression for some intermediary time points, we filtered out genes known to be subject to circadian regulation (a set of approximately 600 genes that were classified as circadian-regulated and varied in expression by 2-fold or more 24–68 h after the transfer of seedlings into continuous light) (Covington and Harmer, 2007). Similarly, we also filtered out a set of approximately 600 transcripts that were found to be significantly differentially expressed (by 2-fold or more) between our 1.5 and 24 h control. A complete list of these excluded transcripts is presented in Table S1. In order to further limit the effects of diurnal regulation, we restricted the majority of our figures to include data from only the 1.5 to 24-h time points after IR treatment. More details are provided in the Materials and Methods.

**Figure 1:**
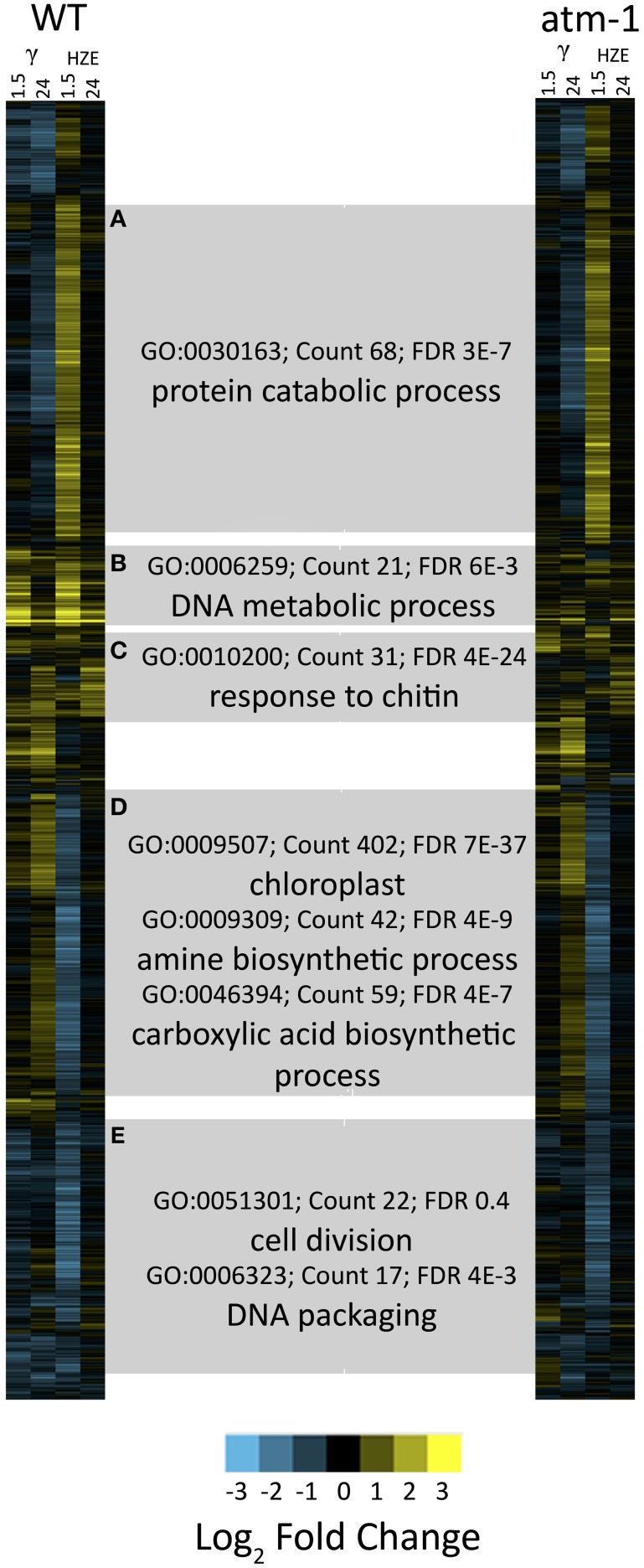
**Clustered expression patterns display coordinated responses to gamma and HZE radiation**. For the set of all transcripts moderately induced/repressed at 1.5 or 24 h after IR treatment (fold change >2 or <-2 and adjusted *p*-value < 0.05 in response to HZE or gamma radiation, for WT or *atm-1* plants), we display clustered expression profiles. Each column indicates a particular experimental condition (combination of stress, time point, and spatial region). The profiles are clustered (row clustering) only in terms of the expression values at 1.5 or 24 h after IR treatment. Horizontal gray bars indicate the selection of transcripts in each cluster **(A–E)**. Labels represent significant enrichment for selected GO terms (see text).

The dendrogram in Figure 1 represents all transcripts induced or repressed with 2-fold change and an adjusted *p*-value < 0.05 in either IR treatment at either the 1.5 or 24-h time points. We clustered these transcripts, via xcluster, by their expression values at both time points after irradiation, in WT and *atm-1* plants, and then we visualized their expression profiles across the same set of time points. We also provide a Supplementary Figure (Figure S6) that extends the expression profiles in Figure 1 to include the middle time points (3, 6, and 12 h after IR treatment). Here we present an overview of the major regulatory clusters in Figure 1, their induction by specific agents, and their regulation by ATM. These observations will be discussed in more detail in Results sections Responses Shared by HZE and Gamma Radiation and Comparison of the Transcriptional Response to HZE with that Induced by Other Stressors.

Cluster D represents 1336 transcripts *repressed* by HZE (but not by gamma radiation) in an ATM-independent manner. Among these transcripts, the GO category “Chloroplast” (GO:0009507~chloroplast) is highly overrepresented (FDR 7e-37, 411 genes). This same cluster is also significantly repressed for transcripts involved in both amine and carboxylic acid “biosynthetic processes” (GO:0009309~amine biosynthetic process, FDR 4e-9, 42 genes; GO:0046394~carboxylic acid biosynthetic process, FDR 4e-7, 59 genes). The uniquely HZE-*induced* Cluster A representing 1357 transcripts is overrepresented in “protein catabolic process” (FDR 3e-7, 68 genes). This cluster of induced transcripts follows the same time course as cluster D (see also Figure S6), and like D, is ATM-independent. This suggests that the two clusters (including suppression of amino acid biosynthesis, suppression of protein synthesis, and the induction of degradation of protein) may represent a coordinated response to a single stimulus. In section Results, we revisit this HZE-specific association with protein catabolism.

Cluster C (349 transcripts) of later-induced, largely ATM-dependent transcripts has its peak expression around 6 h for HZE (Figure S6), while its expression is apparent only at 24 h after gamma radiation. This cluster is overrepresented in “response to chitin” (GO:0010200~response to chitin, FDR 4e-24, 31 genes), and the “defense response” (GO:0006952~defense response, FDR 2e-9, 50 genes) both of which overlap strongly with “programmed cell death” (GO:0012501~programmed cell death, FDR 0.07, 14 genes).

Cluster B consists of 424 rapidly induced ATM-dependent transcripts that peak at 1.5 h (the first data point taken) for both types of radiation. These are overrepresented for “DNA metabolic process” (GO:0006259~DNA metabolic process, FDR 6e-3, 21 genes). This cluster contains the most highly induced set of transcripts.

Cluster E (1248 transcripts) includes the suppression of cell-cycle progression-related transcripts (GO:0051301~cell division, FDR 0.4, 22 genes; GO:0006323~DNA packaging, FDR 4e-3, 17 genes). The suppression of these transcripts occurs in response to Gamma radiation and (to a greater degree) in response to HZE. This suppression appears to be ATM-dependent in response to gamma radiation but less so in response to HZE. This cluster is discussed in greater depth in section Effects on Cell Cycle Progression. Previously published work with Gamma-irradiated mutants indicates that cell cycle arrest may be induced either by ATM or by ATR (Culligan et al., 2006).

#### Gamma radiation, HZE, and bleomycin/MMC (“Genotoxic Stress”) induce an ATM-dependent DSB response that is not observed in plants treated with conventional stressors

IR, at these doses, is a stress not found in the natural environment, and it is unlikely that plants have evolved a response to IR *per se*. The transcriptional responses observed here and elsewhere (Culligan et al., 2006; De Schutter et al., 2007; Ricaud et al., 2007) must represent a response to a class of damage (i.e., DSBs, or oxidized cellular components) that is also generated by a more “conventional” stressor (biotic or abiotic), or via an endogenous genomic stress (such as the induction of breaks in meiosis, or transposable element activity). For this reason we compared the transcripts induced by both forms of IR with previously published data describing the transcripts induced by a variety of other abiotic stresses, plus one biotic stress (infection by *Pseudomonas syringae*). Our results are presented in **Figure 6**. This heatmap visualizes (via treeview) the clustered (via xcluster) expression profiles of the set of all transcripts induced 1.5 or 24 h after IR treatment (with log_2_ fold change >2 and adjusted *p*-value < 0.05 in Gamma radiation or HZE, and in WT or *atm-1* plants) across a variety of environmental challenges (Kilian et al., 2007; Usadel et al., 2008; Cools et al., 2011; Zheng et al., 2012; De Coninck et al., 2013) (**Figure 6**, additional information about each of the experimental conditions is presented in Table S3). While there is very little overlap between the effects of IR and that of most conventional stresses, Gamma radiation and HZE share a strong ATM-dependent overlap with “Genotoxic Stress” (simultaneous treatment with both the DSB-inducing agent Bleomycin and the crosslinking agent MMC). ATM is a key component of the response to repair double-stranded breaks (Culligan et al., 2006; Ricaud et al., 2007), and so the observed ATM-dependent overlap of the transcriptional responses to these three DSB-inducing agents probably reflects a response to DSBs *per se*, rather than collateral damage specific to each agent. This figure also reveals some induction- perhaps simply to a lesser degree, of the DSB response by the DNA damaging agents UV-B and hydroxyurea. However, **Figure 6** does not reveal a conventional stress (i.e., drought, cold, infection) that provokes this “DSB response.”

**Figure 2:**
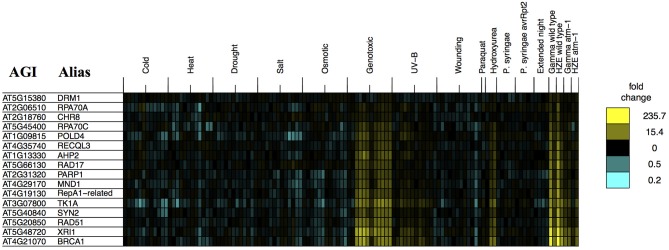
**Stress profiles of IR-induced DNA metabolism transcripts**. For all transcripts in GO: “DNA metabolic process” that are significantly induced (adjusted *p*-value < 0.05, fold change >2) 1.5 h after both HZE and Gamma radiation, in WT plants, we display expression profiles across all abiotic and biotic stresses. Each column indicates a particular experimental condition (combination of stress, time point, and spatial region). We only display the 1.5 and 24-h time points for our experiments with HZE and Gamma radiation.

The analysis described above (**Figure 6**) provides a general overview that makes it easy to visualize major similarities between general responses to IR and various other stresses. In order to determine whether *any* of our queried stresses induce specific transcripts known to be involved in DSB repair, we took the set of transcripts from the “DNA Metabolic Process” GO category that were induced in the early response to both forms of IR (Table 1) and searched for their induction by other stresses (Figure 2). We found that these specific, largely HR-related transcripts were induced by UV-B and hydroxyurea (an inhibitor of dNTP synthesis). Both of these agents are known to induce replication blocks. Replication blocks can be repaired via homologous recombination and can lead to the formation of one-ended DSBs, which also must be repaired by HR. For this reason it is not surprising to find that these agents induce HR-related transcripts.

**Table 1:** **Gamma radiation and HZE share an overrepresentation of DNA metabolic transcripts at 1.5 h after IR**.

**agi**	**GW**	**HW**	**GA**	**HA**	**Description**
At4g21070	7.4	7.9	3.3	0.6	BRCA1 ubiquitination, transcription, cell cycle
At5g48720	7.0	7.1	2.5	1.0	XRI1 x-ray induced 1 interacts with MND1
At5g20850	5.3	5.4	1.6	0.3	RAD51 homology search/base pairing during HR
At5g40840	4.8	5.3	1.2	0.2	SYN2: sister chromatid cohesion 1 homolog 2
At3g07800	4.3	4.3	1.8	−0.2	TK1A Thymidine kinase 1A
At4g19130	4.2	4.8	0.8	−0.1	RPA1E replication factor-A protein 1-related (Aklilu et al., 2014)
At4g29170	3.9	4.4	0.4	−0.1	Mnd1: interacts with AHP2 in synapse formation
At2g31320	3.6	3.9	0.4	0.0	PARP1 poly(ADP-ribose) polymerase 1
At5g66130	3.6	5.0	0.5	0.2	RAD17
At1g13330	3.2	4.0	0.4	−0.1	AHP2 Hop2 homolog
At4g35740	3.1	4.1	0.2	0.6	RecQl3 helicase
At1g09815	3.0	2.6	0.4	−0.2	POLD4 polymerase delta 4
At5g45400	2.3	1.8	0.3	−0.6	RPA70C/RPA1C replication factor-A protein 1-related
At2g18760	1.4	2.1	0.2	0.3	CHR8 chromatin remodeling 8
At2g06510	1.2	2.6	0.1	0.4	RPA70A/RPA1A replication protein A
At5g15380	1.1	1.2	0.1	0.2	DRM1 domains rearranged methylase 1

*Significantly induced (fold change >2, adjusted p-value < 0.05) transcripts by gamma radiation and HZE at 1.5 h after IR in GO: DNA Metabolic Process. Fold Enrichment is shown under each treatment. G = gamma radiation, H = HZE, W = WT, A = atm-1. Log_2_ fold induction is shown*.

However, it is interesting to see that none of the other biotic or abiotic stresses investigated here invokes the DSB response. We might further speculate, based on this, that these stresses do not induce significant levels of DSBs *or* replication blocks, in spite of the fact that many stresses induce the production of ROS in plants (Suzuki et al., 2012; Choudhury et al., 2013). Strikingly, Paraquat treatment itself, a very potent source of ROS, did not induce the DSB response. Given that there clearly is a programmed DSB response in Arabidopsis, the “DSB response” may reflect an evolutionary adaption to some other DSB-inducing event- perhaps the induction of DSBs by naturally occurring external chemical agents not tested here. Induction of both DSBs and HR-related transcripts has also been observed during the very early stages of seed imbibition (Waterworth et al., 2010), and may reflect the accumulation of these lesions during desiccation and rehydration. Alternatively, DSBs might be induced endogenously, without environmental influences- through the activation of transposable element activity, or the programmed induction of DSBs that occurs during meiosis. However, our results indicate that DSBs, or at least the DSB response, are not induced by the conventional stresses tested here.

We did observe some overlap between IR (but not “genotoxic stress”) and certain abiotic stresses- these are described further in section Comparison of the Transcriptional response to HZE with that Induced by Other Stressors.

### Responses shared by HZE and gamma radiation

#### The shared response at 1.5 h is overrepresented for transcripts related to DNA metabolism

**Figure 3:**
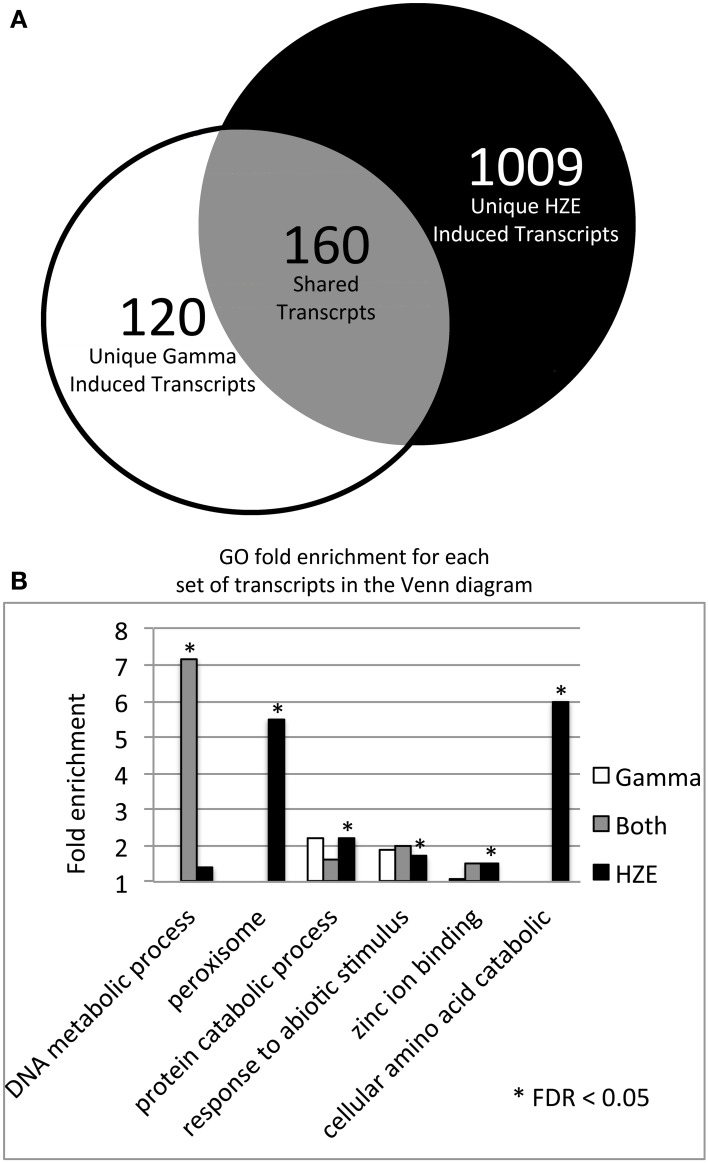
**HZE and gamma induce DNA metabolic transcripts 1.5 h after IR. (A)** The Venn diagram shows the overlap of the 280 gamma radiation and 1169 HZE-induced transcripts at 1.5 h after irradiation (fold change >2 and adjusted *p*-value < 0.05). We used the 1.5 h unirradiated control for the 1.5–12 h time points and the 24 h unirradiated control for the 24 h time point. **(B)** The bar graph shows the fold enrichment of the gamma radiation unique, HZE unique, and shared induced transcript sets shown in the Venn diagram in **(A)** for each GO category that is significantly overrepresented in at least one of these transcript sets.

HZE and gamma radiation induce both singly damaged sites and clustered lesions in DNA, which lead to DSBs. However, our two treatments differ in both the severity of the clustering in DNA and the quantity of the remaining non-clustered damage. A comparison of the transcriptomic effects of the two treatments can help us identify which genes are candidates for the repair of lesions induced by both agents. Thus, we compared the specific set of transcripts induced by HZE at 1.5 h after treatment (adjusted *p-value < 0.05*, at least 2-fold induction) to that induced by gamma radiation, in wild type plants (Figure 3A). We found that of a total of 280 gamma radiation-induced and 1169 HZE-induced transcripts, only 160 transcripts were shared. This is surprisingly small degree of overlap, and suggests that plants irradiated with the two different agents receive significantly different spectra of damage.

Of the 160 shared transcripts, 16 fall into the “DNA Metabolism” GO category (GO:0006259~DNA metabolic process). This is the most overrepresented category among the shared transcripts at this time point (Figure 3B). The majority of these induced DNA Metabolism genes play a role in the repair of DSBs via homologous recombination (Table 1). Other transcripts that we might expect to be induced by IR, such as components required for nonhomologous end-joining, for nucleotide excision repair, or for the base excision repair of oxidized bases, were not found among the shared transcripts, but were observed to be induced (at significant but rather low amplitude) in the HZE-treated plants (Table 2, see section Effects on Cell Cycle Progression for further discussion). This suggests that basal levels of expression for these genes may be sufficient for plants irradiated at this dose of gamma, while the same is not true for HZE.

**Table 2:** **“DNA metabolic response” transcripts induced by HZE, but not Gamma irradiation at 1.5 h after IR**.

**agi**	**Description**	**GW 1.5**	**HW 1.5**	**GA 1.5**	**HA 1.5**
At1g80850	3-mA glycosylase-like, BER	2.0	3.2	0.9	0.35
At5g16630	*XPC*, damage recognition for NER	1.1	2.5	0.81	2.3
At1g02670	P-loop helicase	1.2	4.9	0.87	1.5
At2g13840	DNA polymerase-like	0.87	2.3	0.93	2.1
At1g30480	*DRT111*, HR	1.74	2.5	1.1	1.5
At1g49980	Y-family polymerase, *dinB* like	1.4	2.6	1	1.2
At3g02540	*RAD23-3*, ubiquitination	1.1	3.5	1	2.8
At4g36050	Endo/exonuclease family	1	2.6	0.87	3.2
At2g30350	*uvrC*-like, organellar NER?	0.87	3.2	0.7	3.0
At5g14620	*DMT7/DRM2* DNA methyltransferase	1	2.6	0.7	1.5
At5g57160	*LIG4*, NHEJ	1.9	2.3	0.87	1.3
At1g80420	*XRCC1*, BER, DNA demethylation	0.57	4	0.81	3.7
At4g31150	Endonuclease V family	0.93	2.3	0.87	1.9
At3g12710	3-mA glycosylase-like, BER	1.1	2.1	0.66	1.5
At5g58720	*SMR* (Small MutS Related)	0.93	2.8	1	2.3

*The 15 transcripts are observed among the 1009 transcripts that are significantly induced (fold change >2, adjusted p-value < 0.05) at 1.5 h after treatment by HZE (HW1.5) but not Gamma radiation (GW1.5). Log_2_ fold induction or repression is shown. G = gamma radiation, H = HZE, W = WT, A = atm-1*.

#### Human homologs of transcripts induced by both forms of IR

Transcriptional induction provides clues to the recruitment of proteins required for a given cellular process. While many different metabolic processes may be induced in response to HZE and gamma radiation, a DSB repair is clearly a shared response (Table 1). Taking advantage of the high conservation of DNA repair proteins among eukaryotes, this dataset may allow us to identify novel DSB response proteins in both Arabidopsis and mammals. Using BLAST, we found that 72 of the 160 shared transcripts induced by both forms of radiation shared homology with human proteins (e-value < 1e-6) (Table S2). Many of these transcripts are known to be involved in DSB repair, DNA replication, DNA methylation, and cell cycle control in humans and Arabidopsis (highlighted). AT5g49110, annotated as an unknown protein in Arabidopsis, was found to be homologous to a human protein annotated as “PREDICTED: Fanconi anemia group I protein.” Fanconi anemia is a genetic disorder linked to defects in DNA repair-particularly the repair of interstrand crosslinks (Kim and D'Andrea, 2012). Our results provide additional support for the prediction that this protein is involved in the DSB response.

#### HZE-treated plants exhibit a greater dependence on ATM for the induction of DSB-repair-related transcripts

**Figure 4:**
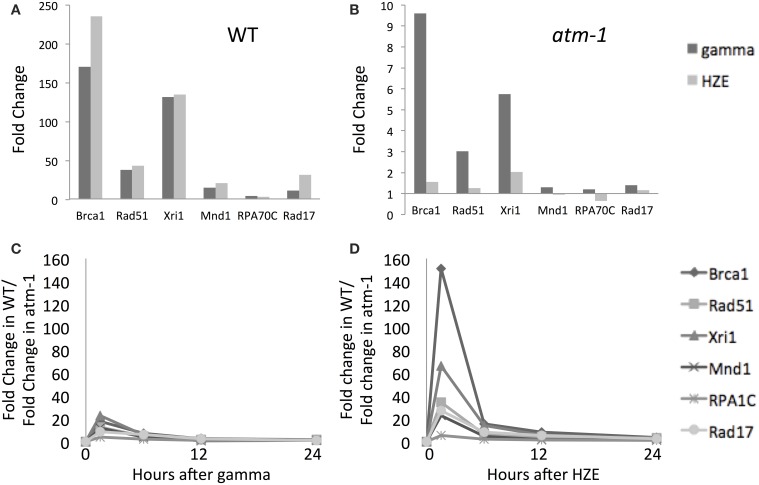
**Both gamma and HZE induce double-strand-break repair transcripts**. Fold change of expression for double-strand-break repair transcripts at 1.5 h after gamma radiation (dark vertical bars) or HZE (light vertical bars) in WT **(A)** or *atm-1* **(B)** seedlings. The ratio between the fold changes of expression for WT and *atm-1* was calculated at 1.5, 6, 12, and 24 h after treatment with gamma radiation **(C)** or HZE **(D)**.

At the doses used here (100 Gy gamma radiation and 30 Gy HZE, which have equivalent effects on root growth) the level of expression of DSB-repair-related transcripts at 1.5 h is of a similar order of magnitude (Table 1, Figure 4A). The fact that HZE treatment deposits less energy but triggers similar levels of induction of these genes may reflect a difference in either the efficiency per Gray of induction (or the time required for repair) of DSBs induced by this agent. In order to determine whether transcript induction increases with the level of damage incurred, we measured the effect of an increasing dose of gamma radiation on the fold-induction of the repair genes *BRCA1* and *RAD51*. We found that the transcriptional response of these two genes does indeed scale up with IR dose (Figure S1). Thus, the level of expression of these characteristic HR genes might be used as a proxy for not only the presence but also the frequency of DSBs.

DSB repair-related transcripts are induced rapidly by both agents (Figure 4A) and this induction is more heavily dependent on ATM in the early time-points (Figures 4B–D). The ATM dependence of the early response suggests recognition and signaling of DSBs. In contrast, the continued expression of these transcripts at 24 h (Figure 2) appears to be completely ATM-independent (Figures 4C,D). Although both gamma and HZE induce DSB repair transcripts to a similar degree (Figure 4A) this induction is far more ATM-dependent in HZE-irradiated cells (Figures 4C,D). This suggests that ATR may play a more important role in activation of DSB repair in gamma-irradiated plants than it does in HZE-irradiated plants. It is possible that gamma radiation triggers more replication stress than HZE, thus activating an ATR-dependent (rather than ATM-dependent) DNA repair pathway. We discuss this possibility below.

#### Effects on cell cycle progression

Cyclin transcripts are similarly suppressed by both gamma radiation and HZE at 1.5 h after irradiation. This presumably reflects an the suppression of cell cycle progression. This effect is largely, but not entirely, alleviated by 24 h post-IR (Figure S4). *CycB1;1*, an exceptional cyclin known to be highly induced by gamma radiation (Culligan et al., 2006) is also induced by HZE (Figure S4).

**Figure 5:**
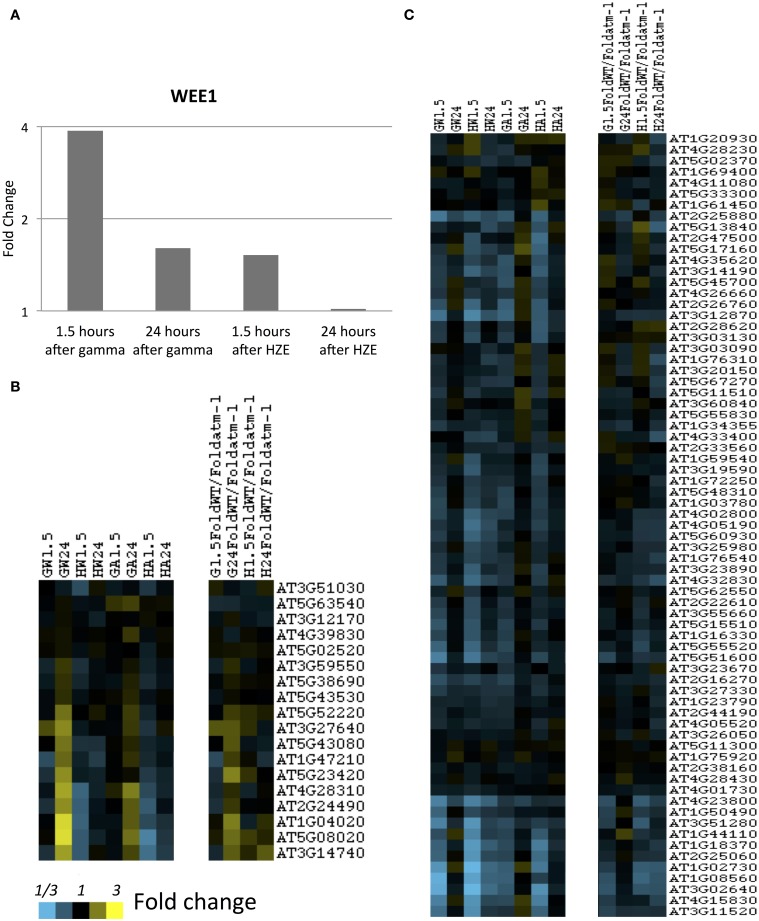
**S-and-M-phase-specific transcripts at 1.5 and 24 h after gamma or HZE**. **(A)** Fold change of expression of Wee1 was calculated at 1.5 and 24 h after IR. Fold-change of expression was also calculated for **(B)** S-phase specific, or **(C)** M-phase specific transcripts (Menges et al., 2005) at 1.5 and 24 h after gamma radiation in WT (GW1.5 and GW24), 1.5 and 24 h after HZE in WT (HW1.5 and HW24), 1.5 and 24 h after gamma radiation in *atm-1* (GA1.5 and GA24), and 1.5 and 24 h after HZE (HA1.5 and HA24). In addition, we reported the ratio between the fold changes of expression for WT and *atm-1* at 1.5 and 24 h after treatment by HZE or gamma radiation.

In order to determine whether cells were accumulating in a particular phase of the cell cycle, we looked at expression levels of transcripts associated with S or M phase (identified in sucrose-starved synchronized Arabidopsis cells) (Menges et al., 2005) (Figures 5B,C). As we see in Figure S5, both gamma radiation and HZE-treated seedlings exhibit suppression of transcripts associated with both M or S phase, indicating that neither phase of the cell cycle is progressing normally. By 24 h, HZE-treated seedlings are slightly repressing some transcripts associated with S or M phase, but nearly back to unperturbed levels of expression. In contrast, at 24 h gamma-irradiated seedlings have begun to hyperexpress many S phase associated transcripts (in a partially-ATM-dependent manner) (Figure 5B), suggesting that, at 24 h, cells of gamma irradiated plants may be overrepresented for S phase. It is possible that this reflects the accumulation of cells, at earlier time points, at an S- or intra-S checkpoint.

The notion that gamma-irradiated seedlings are undergoing replication stress is also consistent with our observation of the stronger induction of Wee1 in gamma-irradiated plants vs. HZE-irradiated plants, at 1.5 h (Figure 5A). *WEE1* is protein kinase involved in adaptation to replication stress in plants (Cools et al., 2011) which is induced during S phase in the HU-stressed cell, allowing it to proceed through S phase without an extended delay, and preventing the premature cellular differentiation that is observed in permanently arrested meristematic cells. The effects observed on cell-cycle related transcripts are muted, but not entirely absent in the *atm-1*mutant.

The difference in expression of the above markers is consistent with the, hypothesis that gamma radiation is inducing a replication stress not incurred by HZE-treated cells. It should be noted that approximately 50% of the energy deposited by a 1 GeV/n Fe ion is thought to be deposited with the 9 nm “core” of the particle's path—the remaining 50% of its energy would produce a “penumbra” of high-energy electrons similar to those produced by gamma radiation (Magee and Chatterjee, 1980). Given that we have applied 30 Gy of HZE vs. 100 Gy of gamma radiation, we would expect that relatively little (15 Gy vs. 100 Gy) of the Fe ion's energy is deposited in the “dispersed clusters” characteristic of gamma radiation rather than along the path of the particle. In short, the amount of HZE-induced damage to DNA not within the core radius of the particle would be expected to be only 15% that induced by gamma radiation. Thus, gamma radiation, at 100 Gy, may be generating more replication blocks than HZE at 30 Gy. While DNA in the path of the HZE particle's core radius is extensively damaged, and this damage is difficult to repair, a replication block is still a replication block regardless of the multiplicity of lesions at a particular site.

#### A set of HZE-specific induced transcripts involved in DNA metabolism

While a large number of genes in the “DNA metabolic process” GO category are induced in response to both HZE and gamma radiation at the 1.5-h time point (Table 1), an additional 16 genes in this category are induced in response to HZE but not Gamma radiation at the same time point (Table 2). These transcripts are known (LIG4) or predicted to participate in a variety of DNA-related processes (BER, NER, NHEJ, DNA methylation) and vary in their dependence on ATM. Also at 1.5 h, we observe a large number of transcripts induced by HZE but not Gamma; these are ATM-independent. We will discuss this general response in the next section.

### Comparison of the transcriptional response to HZE with that induced by other stressors

#### Visualization of transcriptomic overlaps between ionizing radiation and other abiotic and biotic stresses

GO category overrepresentation is a useful way to sift through transcriptomics data in the hope of identifying the metabolic nature of the response to a given stress. While we made use of GO enrichment throughout our own analysis, we acknowledge that it has its limitations. Significant enrichment of a GO category is not a guarantee of the activation of a particular biological process, nor will it reveal the reason for which that process is activated. A complementary approach to understanding the biological processes activated by a given stress is to consider the overlap between the transcriptional response under scrutiny and published responses to other stresses. If there is significant overlap between the transcripts induced by IR and those induced by a different, more thoroughly studied stress, then we can leverage our understanding of the plant's response to the other stress in order to make inferences about IR. This approach may be particularly useful when investigating an “unnatural” stress such as high dose rate IR. We would expect the plant to lack evolved responses specific to IR and thus be limited to “sampling” the programmed responses to natural stresses, according to similarities in the inflicted damage.

**Figure 6:**
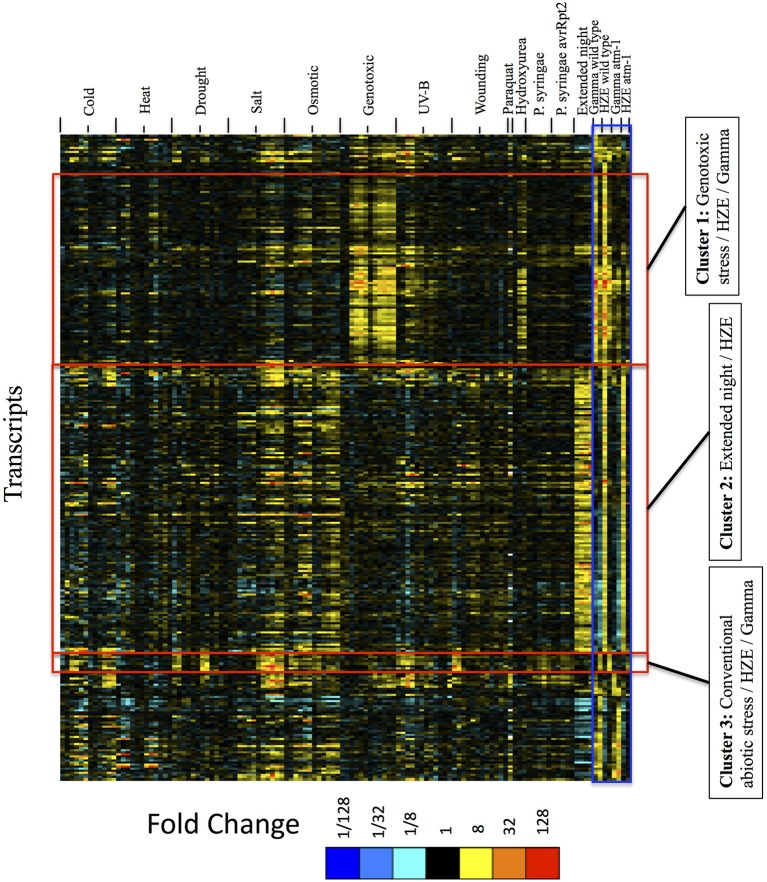
**Expression profiles of IR-induced transcripts, across abiotic and biotic stresses, clustered by the expression profiles across IR experimental conditions only**. For the set of all transcripts strongly induced at 1.5 or 24 h after IR treatment (fold change >4 and adjusted *p*-value < 0.05 in response to HZE or gamma radiation, for WT or *atm-1* plants), we display expression profiles across all abiotic and biotic stresses. These profiles are clustered (row clustering) only in terms of the expression values at 1.5 or 24 h after IR treatment. Each column indicates a particular experimental condition (combination of stress, time point, and spatial region). We only display the 1.5 and 24-h time points for our experiments with HZE and Gamma radiation.

With this goal in mind, we considered the extent to which the transcriptional responses to HZE and gamma radiation resemble the responses to a range of different abiotic and biotic stresses. To test for shared transcriptional responses, we visualized (via treeview) the clustered (via xcluster) expression profiles of the set of all transcripts induced at 1.5 or 24 h after IR treatment (with log_2_ fold change >2 and adjusted *p*-value < 0.05 in Gamma radiation or HZE, and in WT or *atm-1* plants) and compared these to a transcript sets induced by a wide variety of abiotic and biotic stresses (Figure 6). Specifically, we first clustered these transcripts across their IR-induced expression profiles, and then we extended the profiles to include the expression patterns across the abiotic and biotic stress conditions. The IR-induced transcripts fall roughly into several clusters, three of which are consistent with a shared programmed response between HZE and (1) DSB-inducing agents, (2) extended night, and (3) multiple conventional stresses. Figure S3 displays the corresponding clustered expression profiles for all IR-repressed transcripts, using the same cutoff parameters.

The results from Figure 6 suggest that seedlings subjected to DSB-inducing Gamma radiation or radiomimetic chemicals- to an extent that transiently inhibits growth- induce few of the transcripts commonly expressed by seedlings subjected to other abiotic stresses. Thus, the response to these DSB-inducing agents appears to be relatively unique. However, treatment with HZE (but not gamma radiation or “genotoxic agents”) did induce some transcripts that are similarly induced by “Extended night” (Cluster 2 of Figure 6). The term “Extended night” refers to the plant's response to the extension of the length of night- via a delayed dawn, rather than an early sunset (Usadel et al., 2008). This similarity between extended night and HZE response is limited to early time points (1.5 and 3 h) after HZE treatment, but includes all reported time points of extended night. This “Extended night-like response” induced by HZE is not ATM-dependent (Figure 6), and, again, is not observed in gamma radiation or BLM+MMC-treated plants- strongly suggesting that this response is not the result of DSB induction, but is instead due to some other lesion(s) induced specifically by HZE.

Treatment with HZE and Gamma also induced a set of transcripts (absent in “genotoxic agents”) that are similarly induced across a wide variety of conventional stresses (Cluster 3 of Figure 6: cold, drought, salt, osmotic, UV-B, and wounding). Here, we define “conventional” as stresses that we perceive to be more commonly occurring in nature. This similarity is limited to the late response to HZE and Gamma (24 h) but is more clearly observable in the early responses to conventional stresses (0.5–6 h). The fact that this shared response is dependent on the presence of ATM in the IR-treated plants is surprising given the fact that, in plants, ATM is known exclusively for its role in the response to DSBs. Extending the profiles of transcripts in Cluster 3 (IR + multiple conventional abiotic stresses) to include all time points for IR treatment shows strong induction at 6 and 12 h after HZE but not Gamma radiation, suggesting that this particular set of transcripts is induced earlier in response to HZE vs. gamma radiation (Figure S2). The relative strength of the IR signal vs. the circadian and diurnal time series suggest to us that any circadian or diurnal bias at the middle time points would not affect our overall conclusions. We have extended Figure S2 to include previously published circadian and diurnal time series profiles in order to aid the reader in drawing their own conclusions.

The fact that transcripts with similar expression patterns in response to IR also had very similar expression patterns across the other stress conditions suggest that these sets of transcripts are coordinately regulated in response to both IR and the other stresses. We hypothesize that these sets of co-expressed transcripts comprise distinct transcriptional programs that evolved in response to naturally occurring stresses but can also be triggered when unnatural stresses, such as HZE and gamma radiation, induce patterns of damage or signals in the plant that resemble those induced by the naturally occurring stresses. We follow up this hypothesis in the context of the two candidate IR-induced programmed responses shared with extended night and conventional stress in subsections Transcripts Strongly Induced in Response to Extended Night are also Induced in an Early ATM-Independent Transcriptional Response to HZE and HZE Triggers an ATM-Dependent Transcriptional Response that is also Induced by Several Conventional Abiotic Stresses.

#### Transcripts strongly induced in response to extended night are also induced in an early ATM-independent transcriptional response to HZE

Extended night is known to trigger a variety of biological processes, many of them resulting from a shortage of energy stores in the form of carbon. Leaf starch accumulates during the day but most of it is gone by the end of the night (Zeeman et al., 2007; Usadel et al., 2008). In an extended night, the plants respond to lack of stored energy in two ways: (a) by slowing growth and (b) by looking for alternative internal sources of energy. Extended night results in a three-fold decrease in the level of trehalose-6-phosphate (T6P) (Lunn et al., 2006), an important positive regulator of growth (Delatte et al., 2011; Schluepmann et al., 2012), as well as the induction of genes associated with amino acid catabolism (Usadel et al., 2008). In addition, fatty acid beta-oxidation- a process by which lipids are broken down for energy- has been shown to be a key component of the response to extended night (Kunz et al., 2009). In this section, we present evidence that many of the above processes are transcriptionally active in the early response to HZE treatment as well as in the response to extended night.

**Figure 7:**
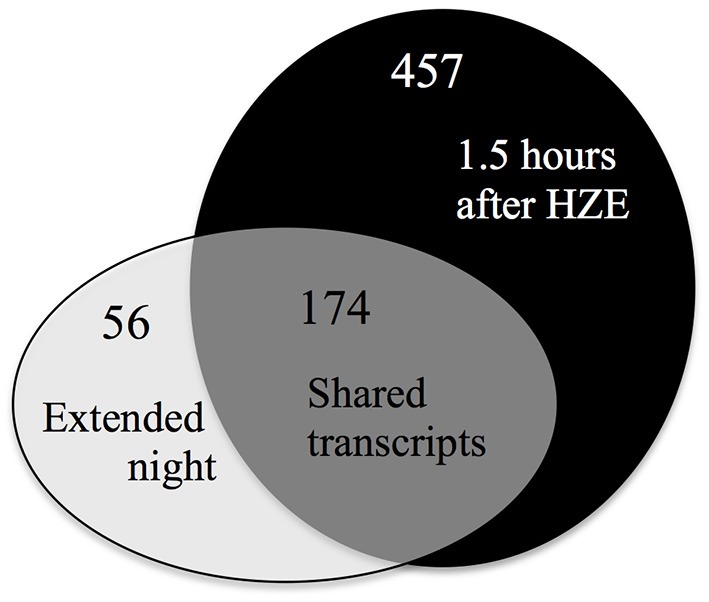
**Most transcripts induced by extended night are also induced by HZE**. Overlaps between up-regulated transcripts (fold change >2 and adjusted *p*-value < 0.05) induced early in the response to HZE treatment (at 1.5 h in both WT and *atm-1*) vs. induced persistently in response to extended night.

To characterize the full extent of the shared induction between these two stresses, we identified commonalities using a fixed criterion for induction, specifically a fold change cutoff of 2 and an adjusted *p*-value cutoff of 0.05. Under these settings, we identified a set of 457 transcripts that were induced at 1.5 h after HZE treatment of wt or *atm-1* plants. Applying the same cutoffs to published data on transcriptional response to extended night, we found 230 transcripts were induced across all four of the time points. There is an overlap of 174 induced genes between these two transcript sets (Figure 7). 83 of transcripts are present in the 136 genes in Cluster 2 of Figure 6, 158 overlap with the 1357 genes in cluster A from Figure 1 (which was described earlier as being overrepresented for the GO category “protein catabolic process”). The fact that the majority of the transcripts persistently induced in response to extended night were also induced in the early response to HZE (Figure 7) suggests that HZE treatment triggers most components of the transcriptional program for response to extended night.

GO enrichment analysis of this set of 174 shared genes suggests that several of the biological processes that are associated with the response to extended night are also transcriptionally activated in the early response to HZE treatment. These processes reflect the catabolism of cellular components, including “fatty acid beta-oxidation” (GO:0006635, 25.0-fold enrichment, FDR 5.9E-2, 5 genes) together with “peroxisome” (GO:0005777, 6.3-fold enrichment, FDR 2.5E0, 6 genes), the peroxisome being a site of fatty-acid beta-oxidation in plants. We also found strong enrichment of “cellular amino acid catabolic process” (GO:0009063, 15.9-fold enrichment, FDR 3.6E-1, 5 genes). These enrichment results suggest that increased catabolism of fatty acids and amino acids is part of the early response to HZE treatment- a response not induced by other DSB-inducing agents.

**Figure 8:**
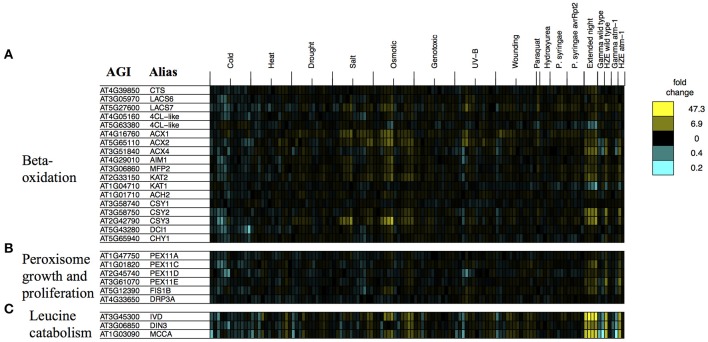
**Stress profiles of transcripts associated with extended night**. For transcripts related to key biological processes that appear to be transcriptionally activated in response to both HZE (early response) and extended night- **(A)** beta-oxidation, **(B)** peroxisome growth and proliferation, and **(C)** leucine catabolism- we display expression profiles across all abiotic and biotic stresses. Each column indicates a particular experimental condition (combination of stress, time point, and spatial region). We only display the 1.5 and 24-h time points for our experiments with HZE and Gamma radiation.

We examined the expression profiles of a number of transcripts associated with beta-oxidation that were compiled in a review study (Baker et al., 2006) (we excluded any transcripts that did not pass our circadian and developmental filtering criteria, which are described in the Materials and Methods). Consistent with the activation of fatty acid beta-oxidation, we observe strong induction of many of the beta-oxidation associated transcripts described in this study (Figure 8A).

Fatty-acid beta-oxidation is known to occur in the peroxisomes (including glyoxysomes), although there is a body of evidence (Masterson and Wood, 2000) suggesting that it also occurs in the mitochondria. Given the proposed increase in fatty acid beta-oxidation under extended night (and HZE) treatment, one might consider whether it would be accompanied by an increase in the number of peroxisomes. The placement of excised leaves of *Pisum sativum* in the dark for 3–11 days results in an increase in the number of peroxisomes (Pastori and Delrio, 1994), suggesting that extended night treatment might have a similar effect on Arabidopsis. We found that a group of transcripts associated with peroxisome growth or proliferation (Lingard et al., 2008) tended to be induced both in the early response to HZE and persistently in response to extended night. Figure 8B illustrates the regulation by stress of genes associated with peroxisome growth (elongation) or proliferation (fission), in one or more of three studies (Lingard and Trelease, 2006; Orth et al., 2007; Lingard et al., 2008). Overexpression of individual PEX11 homologs PEX11a and PEX11c-e has been shown to increase peroxisome elongation and/or duplication; studies disagree on whether the same is true for PEX11b (Lingard and Trelease, 2006; Orth et al., 2007). Subsequent studies suggest that PEX11c-e, FIS1b, and DRP3a all work together to coordinate fission of elongated peroxisomes (Lingard et al., 2008). Again, we found that most of these transcripts (we excluded any transcripts that did not pass our circadian and developmental filtering criteria, which are described in the Materials and Methods) were persistently induced in response to extended night and induced in the early response to HZE (Figure 8B), suggesting that peroxisome growth and proliferation occurs both in the response to extended night and in the early response to HZE. This proliferation may occur to facilitate beta-oxidation of fatty acids.

We also tested the expression profiles of a set of co-expressed transcripts associated with leucine degradation that were compiled by Mentzen et al. (2008) (we excluded any transcripts that did not pass our circadian and developmental filtering criteria, which are described in the Materials and Methods). For all tested transcripts, we found strong induction in the early response to HZE as well as strong, persistent induction in response to extended night (Figure 8C).

While the causal basis for the exceptional similarities between the transcription response to extended night and the early ATM-independent response to HZE remain obscure, we can offer two hypotheses: (a) HZE-treated cells are, for some reason, starving. Perhaps this form of radiation, at this intensity of dose, disrupts mitochondrial or chloroplast function? or (b) The extensive induction of catabolic processes for both lipids and proteins might reflect the degradation of damaged cellular components.

#### HZE triggers an ATM-dependent transcriptional response that is also induced by several conventional abiotic stresses

As described above, Figure 6 is a dendrogram generated by sorting by the patterns of expression of all transcripts that are induced 1.5 or 24 h after either IR treatment, in WT or *atm-1* plants. This dendrogram was then extended, without further sorting, to include similar data sets from transcriptomics studies of other more conventional stresses. Here we focus on Cluster 3 of this dendrogram- a set of 9 transcripts induced 24 h, but not 1.5 h, after treatment by HZE or gamma radiation. Although the observed induction of these transcripts is clearly ATM-dependent, in response to gamma radiation, the same is only true for half of the transcripts, in response to HZE. All of these 9 genes also belong to Figure 1's Cluster C, a late-expressed, ATM-dependent cluster of 349 transcripts. Like Cluster C, these 9 genes are highly enriched for “response to chitin” (GO:0010200, 27.6-fold enrichment, FDR 4.9E1, 2 genes) and “defense response” (GO:0006952, 5.1-fold enrichment, FDR 6.2E1, 3 genes) (via the GO DAVID enrichment tool). Consistent with the high enrichment of Cluster 3 for the GO categories for “defense response” and “response to chitin,” we found that a large percentage of these 9 induced genes were induced in response to *P. syringae*, a much larger percentage than for the rest of the strongly IR-induced transcripts. However, this cluster of genes was more strongly induced by a wide variety of stresses, most noticeably cold, salt, wounding, and UV-B.

The (partial) ATM-dependence of the shared response to IR and conventional abiotic stress described above suggests that ATM might possibly play a role in triggering this suite of transcripts in response to conventional abiotic stress treatments. This would be surprising, given that ATM is only known in plants for its role in the response to DNA damage. But such a hypothesis may be consistent with the observations in animal systems for which ATM has been shown to be activated by not only DSBs, but also stimuli such as ROS (Guo et al., 2010) and chromatin hyper-acetylation (Sun et al., 2005; Kaidi and Jackson, 2013). Another hypothesis for the ATM-dependence of this response, which might not necessarily negate the first, is that IR induces some effect on the cell that, in the absence of ATM, derails the plant's normal course of recovery and so results in a suppression of the observed conventional stress program. Such an effect could involve IR-specific patterns of DNA damage, which ATM could counter by its role in the processing and repair of DSBs.

## Conclusions

In this paper, we compare the transcriptomic response to HZE vs. those of other DSB-inducing agents, and then compare that to previously published data sets describing response to a variety of conventional stresses. Some subtle differences were observed between gamma radiation and HZE (section Responses Shared by HZE and Gamma Radiation) which can probably be ascribed to differences in the quantity or quality of DSBs generated by each agent; HZE-generated breaks are expected to be more complex and thus more difficult to repair.

More interestingly, comparison of 3 DSB-inducing treatments: gamma radiation, IR, and a combination of Bleomycin and Mitomycin C (a crosslinking agent), vs. a wide variety of conventional stresses shows that the response to these DSB-inducing agents is a unique “DSB response”- it is very robust, intense, of rather short duration (less than a day), and it is *not* induced by conventional stresses. Thus, this is not a generic response to stress, but a response to a specific lesion in DNA. It has been suggested that DSBs might be generated by conventional stresses in plants, as a wide variety of stresses are known to induce ROS. However, we see here that treatment with Paraquat, a notorious source of ROS that shunts electrons from donors (such as NADPH or Photosystem I) directly to oxygen to produce superoxide does not induce the “DSB response.” Careful observation of the transcripts induced in the DSB response (cluster B, Figure 1) indicates that two other stresses- UV-B and HU, already identified as DNA damaging agents, also induce the DSB response, though at low amplitude, suggesting that these agents induce DSBs at some low frequency (Figure 2).

HZE and gamma radiation both displayed some overlap with a set of transcripts induced by a variety of conventional stresses, particularly at later time points (Figures 6 and S2). These may reflect downstream effects of stress on plant cells, rather than similarities of the immediate effects of each of these stressors. It is interesting that these late-time point commonalities are partially ATM-dependent in HZE and completely ATM-dependent in Gamma (ATM-dependence has not been tested in other stresses). It is somewhat surprising that the shared stress response would be partially or completely eliminated by a defect in ATM- one would guess that a defect in DDR would enhance the stress induced by DNA damage. We should bear in mind, however, that stress response, at least in plants, should not be seen as a set of counterproductive actions resulting from a breakdown of cell function. What appear to be toxic effects of IR [for example cell death (Fulcher and Sablowski, 2009; Furukawa et al., 2010)] are actually ATM-governed orderly responses to DSB-inducing agents. It will be interesting to learn more about the regulators of these “shared stress response” transcripts.

A remarkable similarity was observed between ATM-*in*dependent transcriptional response to HZE and the response to extended night. The extended night response in plants is a response to lack of sugar- leaves store away just enough carbohydrates to make it through to dawn. It seems unlikely that HZE-treated cells are starving at 1.5 h after irradiation, but it is possible that HZE treatment has rendered the energy-producing organelles dysfunctional. The starvation response involves the cannibalization of non-carbohydrate cellular components (proteins and lipids), and we do observe up-regulation of these pathways in HZE-treated plants. It is also possible that the cells are not actually starved, but are instead recycling damaged cellular components.

Do our results inform our understanding of space radiation biology? Yes and no. The identification of a set of transcripts very specifically induced by DSB-inducing agents, and the overrepresentation of repair factors among this set, suggests that the remaining genes of unknown function will also be enriched for this process. Given that many of these have obvious human homologs, this data set undoubtedly includes candidates for previously undiscovered repair functions.

The relevance of the observed HZE-specific responses to space radiation biology are more obscure given the very high dose rate applied here. We ascribe these responses to collateral damage to non-DNA components of the cell, but both our dose (approximately 100× that predicted for a mission to Mars) and dose rate (received in 4 min rather than 4 years) are very high. At more realistic doses damage to membranes and proteins may be slight enough that up-regulation of enzymes that promote “recycling” is not required. On the other hand, it is possible that a single HZE track may generate sufficient damage to provoke such a response- at our dose (30 Gy) we estimate that about 65 particles crossed the nucleus. Given the 30× larger amount of DNA in a human cell, the equivalent amount (though not concentration) of damage would be generated by only 2 or 3 HZE tracks. A recently published transcriptomics study using human fibroblasts and employing a maximum dose of 1 Gy of 1 GeV Fe nuclei (an equivalent dose to ours, if corrected for genome size) also demonstrated both a shared (with gamma radiation) response focused on what the authors describe as “BRCA1-centric repair” and a unique HZE signature. The unique signature was also overrepresented for transcripts related to “pro-inflammatory acute phase response signaling” (Ding et al., 2013). It is interesting that the HZE-specific response in both plants and in animals is overrepresented- albeit slightly- for disease response.

## Accession numbers

All of our microarray data has been deposited in the Gene Expression Omnibus (http://www.ncbi.nlm.nih.gov/geo) with the accession number [GSEXXXX].

### Conflict of interest statement

The authors declare that the research was conducted in the absence of any commercial or financial relationships that could be construed as a potential conflict of interest.
