# Corrigendum: High atomic weight, high-energy radiation (HZE) induces transcriptional responses shared with conventional stresses in addition to a core “DSB” response specific to clastogenic treatments

**DOI:** 10.3389/fpls.2016.01753

**Published:** 2016-11-21

**Authors:** Victor Missirian, Phillip A. Conklin, Kevin M. Culligan, Neil D. Huefner, Anne B. Britt

**Affiliations:** ^1^Department of Plant Biology, University of California, DavisDavis, CA, USA; ^2^Department of Molecular, Cellular, and Biomedical Sciences, University of New HampshireDurham, NH, USA

**Keywords:** DNA repair, double-strand breaks, transcriptomics, stress, cell-cycle, ionizing radiation, HZE, gamma radiation

Due to an oversight, the placeholder for the accession number for our microarray dataset was not updated in the published article. The correct text for the section “Accession Number” should read:

## Accession numbers

All of our microarray data has been deposited in the Gene Expression Omnibus (http://www.ncbi.nlm.nih.gov/geo) with the accession number [GSE61484].

The authors apologize for this oversight. This does not affect the scientific conclusions of the article in any way.

### Conflict of interest statement

The authors declare that the research was conducted in the absence of any commercial or financial relationships that could be construed as a potential conflict of interest.

