# Supplementary material for: High atomic weight, high-energy radiation (HZE) induces transcriptional responses shared with conventional stresses in addition to a core “DSB” response specific to clastogenic treatments
Source: Front Plant Sci. 2014 Aug 1;5:364. doi: 10.3389/fpls.2014.00364 (PMC4117989; doi:10.3389/fpls.2014.00364)
Supplement: Supplementary file 1 [file Presentation1.PDF]

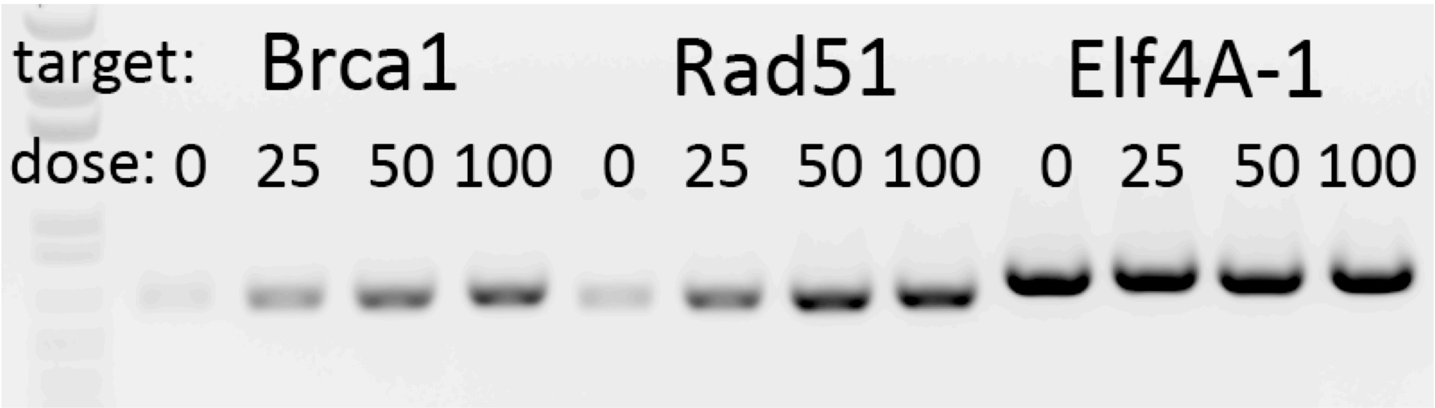

Figure S1. Induced expression of DSB repair transcripts Brca1 and Rad51 scale linearly with dose of IR. Semi quantitative RT-PCR represents transcriptional induction of the DSB repair transcripts Brca1 and Rad51 1.5 hours after 0, 25, 50, 100 Gy gamma IR. Elf4A-1 is used as the loading control target.
