# Supplementary material for: High atomic weight, high-energy radiation (HZE) induces transcriptional responses shared with conventional stresses in addition to a core “DSB” response specific to clastogenic treatments
Source: Front Plant Sci. 2014 Aug 1;5:364. doi: 10.3389/fpls.2014.00364 (PMC4117989; doi:10.3389/fpls.2014.00364)
Supplement: Supplementary file 2 [file Presentation2.PPTX]

## Slide 1
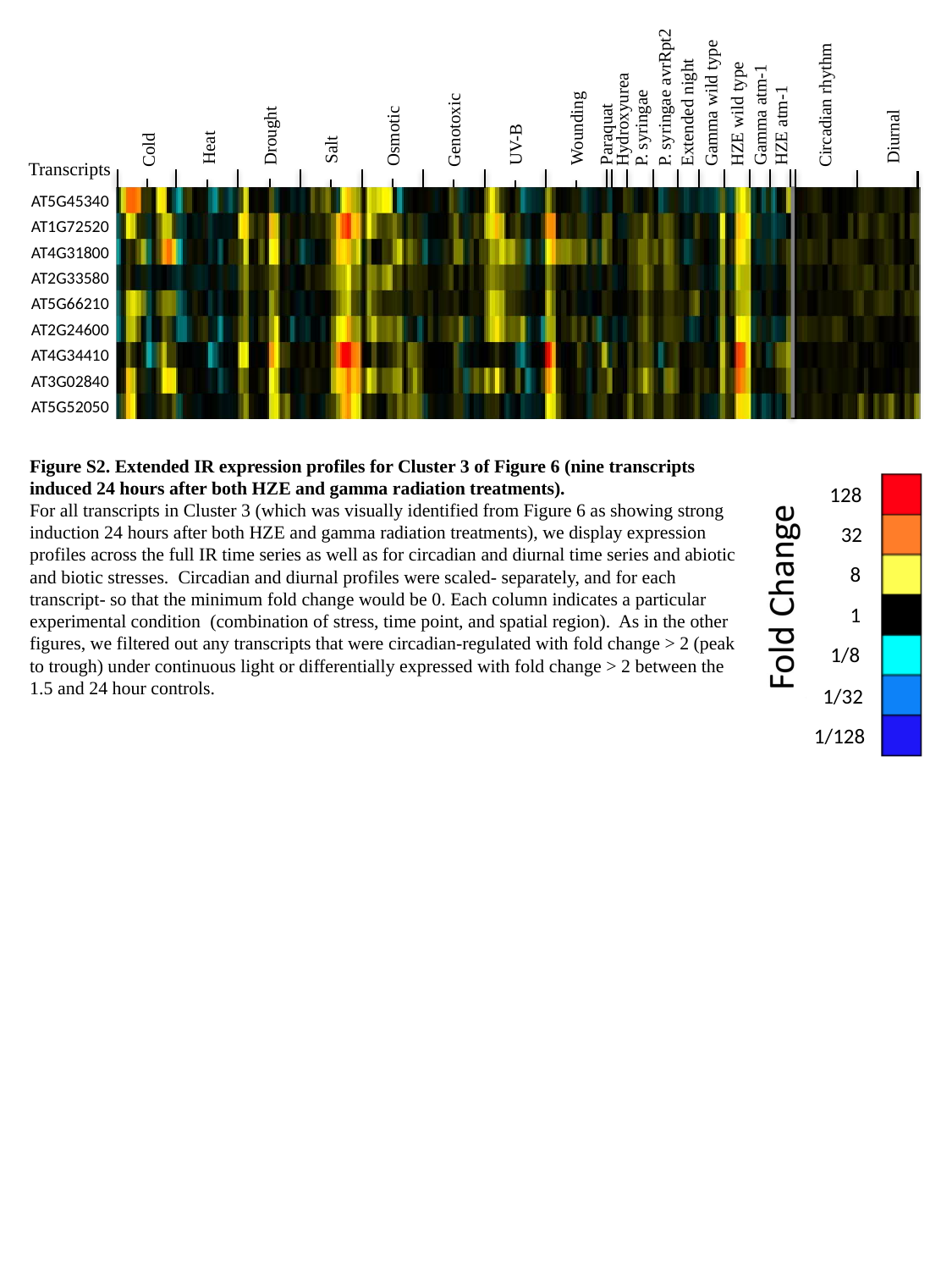

P. syringae avrRpt2
Gamma wild type
Circadian rhythm
Extended night
HZE wild type
Gamma atm-1
Hydroxyurea
HZE atm-1
P. syringae
Wounding
Genotoxic
Paraquat
Osmotic
Drought
Diurnal
UV-B
Heat
Salt
Cold
Transcripts
AT5G45340
AT1G72520
AT4G31800
AT2G33580
AT5G66210
AT2G24600
AT4G34410
AT3G02840
AT5G52050
Figure S2. Extended IR expression profiles for Cluster 3 of Figure 6 (nine transcripts induced 24 hours after both HZE and gamma radiation treatments).
For all transcripts in Cluster 3 (which was visually identified from Figure 6 as showing strong induction 24 hours after both HZE and gamma radiation treatments), we display expression profiles across the full IR time series as well as for circadian and diurnal time series and abiotic and biotic stresses. Circadian and diurnal profiles were scaled- separately, and for each transcript- so that the minimum fold change would be 0. Each column indicates a particular experimental condition (combination of stress, time point, and spatial region). As in the other figures, we filtered out any transcripts that were circadian-regulated with fold change > 2 (peak to trough) under continuous light or differentially expressed with fold change > 2 between the 1.5 and 24 hour controls.
128
32
8
1
1/8
1/32
1/128
