# Supplementary material for: High atomic weight, high-energy radiation (HZE) induces transcriptional responses shared with conventional stresses in addition to a core “DSB” response specific to clastogenic treatments
Source: Front Plant Sci. 2014 Aug 1;5:364. doi: 10.3389/fpls.2014.00364 (PMC4117989; doi:10.3389/fpls.2014.00364)
Supplement: Supplementary file 3 [file Presentation3.PDF]

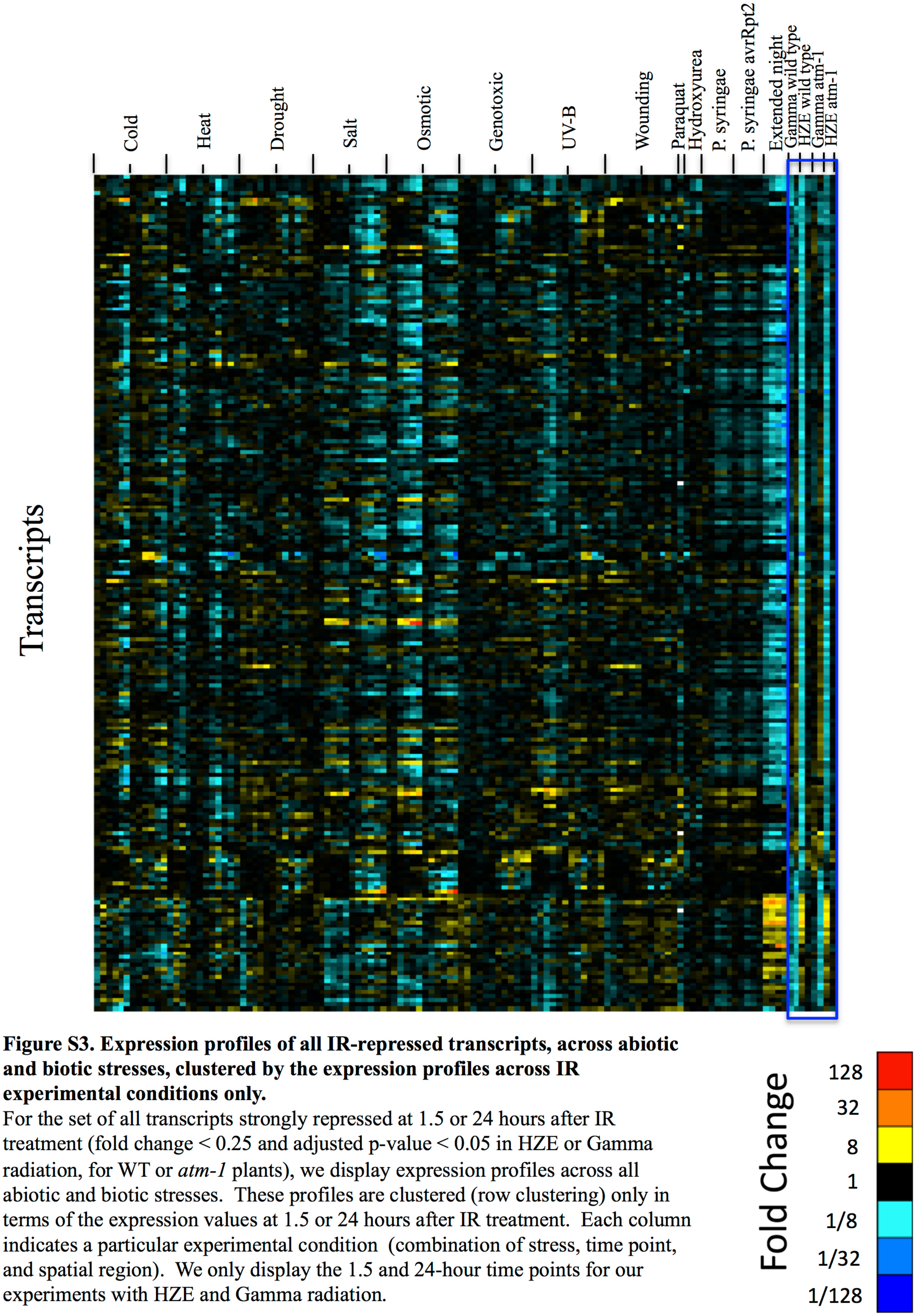

Figure S3. Expression profiles of all IR-repressed transcripts, across abiotic and biotic stresses, clustered by the expression profiles across IR experimental conditions only.

For the set of all transcripts strongly repressed at 1.5 or 24 hours after IR treatment (fold change < 0.25 and adjusted p-value < 0.05 in HZE or Gamma radiation, for WT or *atm-1* plants), we display expression profiles across all abiotic and biotic stresses. These profiles are clustered (row clustering) only in terms of the expression values at 1.5 or 24 hours after IR treatment. Each column indicates a particular experimental condition (combination of stress, time point, and spatial region). We only display the 1.5 and 24-hour time points for our experiments with HZE and Gamma radiation.
