# Supplementary material for: High atomic weight, high-energy radiation (HZE) induces transcriptional responses shared with conventional stresses in addition to a core “DSB” response specific to clastogenic treatments
Source: Front Plant Sci. 2014 Aug 1;5:364. doi: 10.3389/fpls.2014.00364 (PMC4117989; doi:10.3389/fpls.2014.00364)

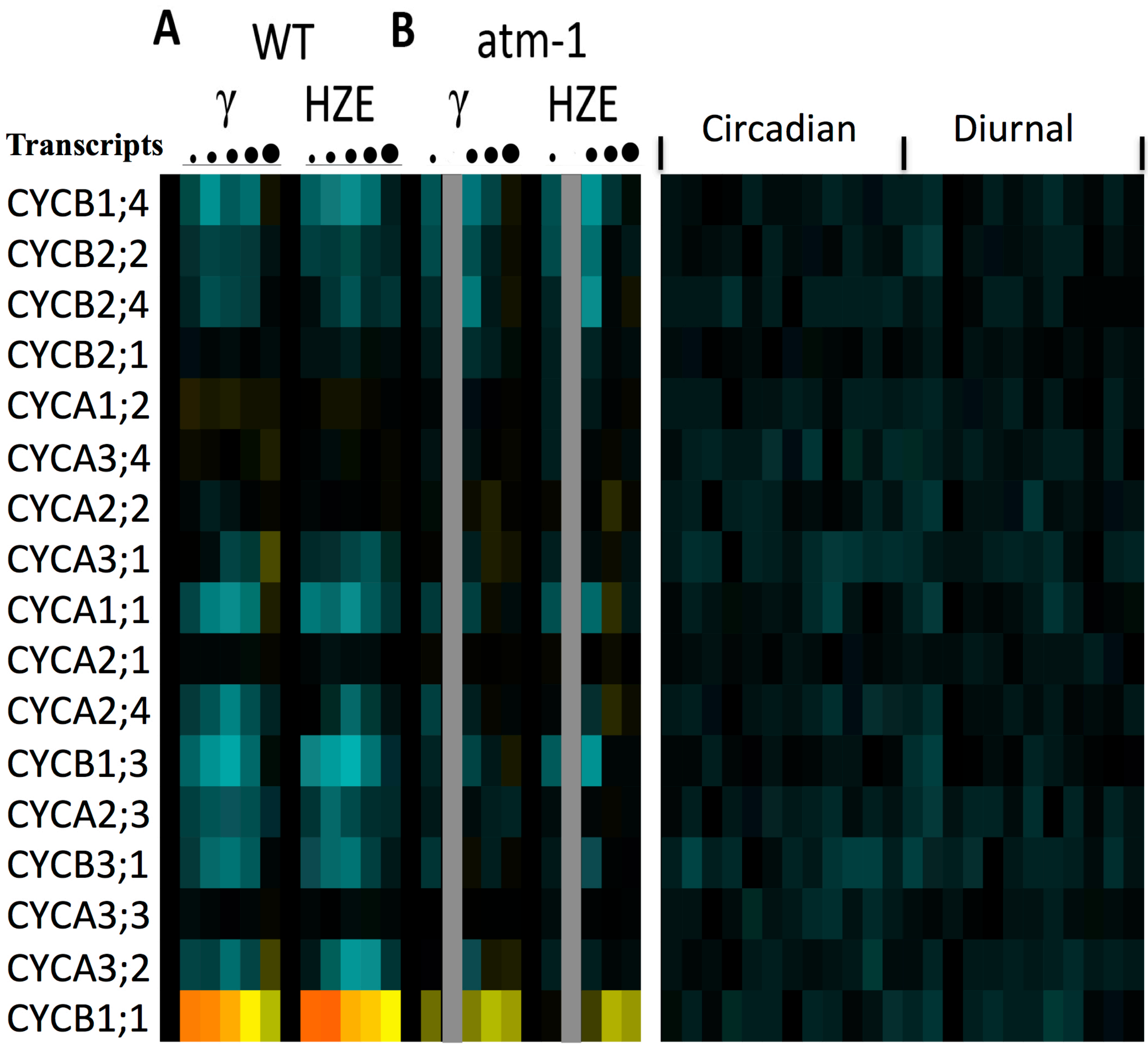

Figure S4. Cell cycle control transcripts indicate a stronger and ATM-dependent delay of cell division in HZE-treated seedlings. Expression profiles, across all time points for HZE or Gamma radiation as well as for circadian and diurnal time series, for Mitotic cyclins in (A) WT seedlings or (B) *atm-1* mutants. Circadian and diurnal profiles were scaled-separately, and for each transcript- so that the maximum fold change would be 0. Each column indicates a particular experimental condition (combination of stress and time point). For each time series, we indicate time points for 1.5, 3, 6, 12, and 24 hours post-irradiation by a series of black dots of increasing size. For the two time series involving an *atm-1* mutant, the 3 hour time point is excluded. We used the 1.5 hour unirradiated control for the 1.5-12 hour time points and the 24 hour unirradiated control for the 24 hour time point. As in the other figures, we filtered out any transcripts that were circadian-regulated with fold change > 2 (peak to trough) under continuous light or differentially expressed with fold change > 2 between the 1.5 and 24 hour controls.

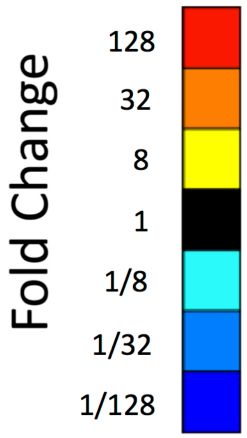

Supplement: Supplementary file 4 [file Presentation4.PDF]
