# Supplementary material for: High atomic weight, high-energy radiation (HZE) induces transcriptional responses shared with conventional stresses in addition to a core “DSB” response specific to clastogenic treatments
Source: Front Plant Sci. 2014 Aug 1;5:364. doi: 10.3389/fpls.2014.00364 (PMC4117989; doi:10.3389/fpls.2014.00364)

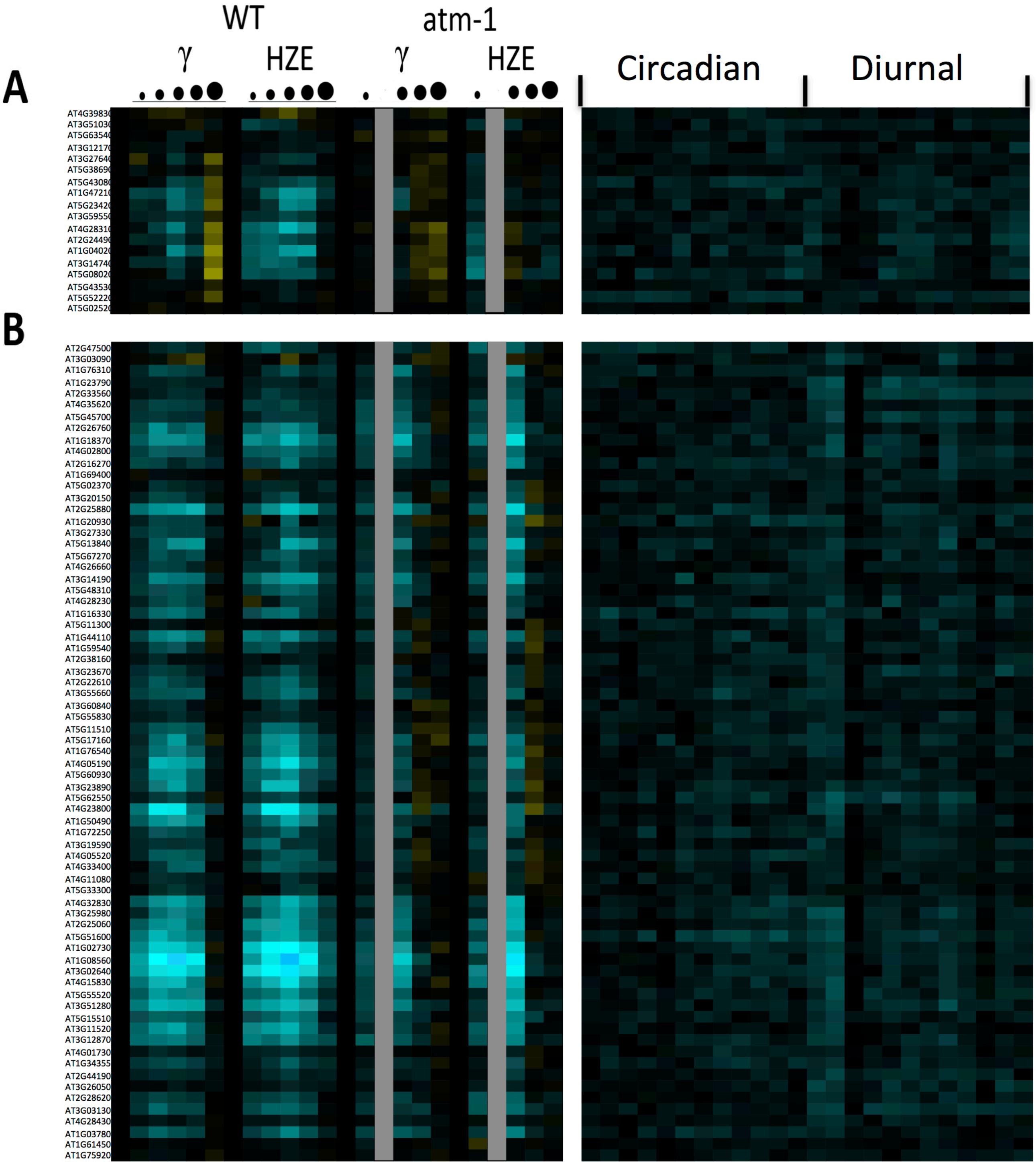


**Figure S5. ATM-dependent-S-and-M-specific transcripts are repressed for a longer period of time under treatment by HZE versus gamma radiation.**
Expression profiles, across all time points for HZE or Gamma radiation as well as for circadian and diurnal time series, for (A) S-phase specific, and (B) M-phase specific genes co-transcribed as in Menges, 2005. Circadian and diurnal profiles were scaled- separately, and for each transcript- so that the maximum fold change would be 0. Each column indicates a particular experimental condition (combination of stress and time point). We used the 1.5 hour unirradiated control for the 1.5-12 hour time points and the 24 hour unirradiated control for the 24 hour time point. As in the other figures, we filtered out any transcripts that were circadian-regulated with fold change > 2 (peak to trough) under continuous light or differentially expressed with fold change > 2 between the 1.5 and 24 hour controls.

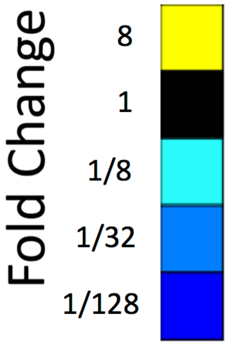

Supplement: Supplementary file 5 [file Presentation5.PDF]
