# Supplementary material for: High atomic weight, high-energy radiation (HZE) induces transcriptional responses shared with conventional stresses in addition to a core “DSB” response specific to clastogenic treatments
Source: Front Plant Sci. 2014 Aug 1;5:364. doi: 10.3389/fpls.2014.00364 (PMC4117989; doi:10.3389/fpls.2014.00364)

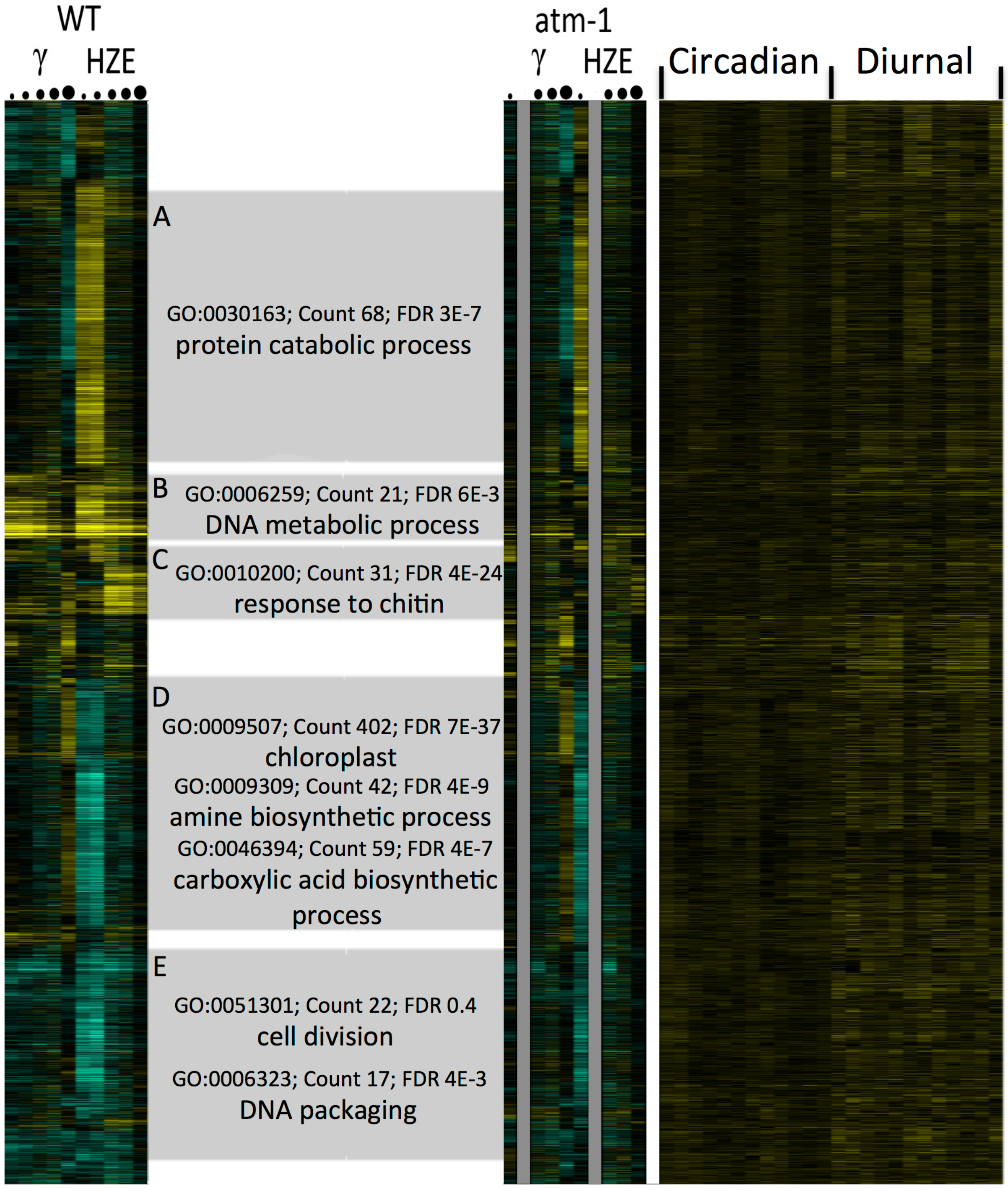

Figure S6. Clustered expression patterns display coordinated responses to gamma and HZE radiation. For the set of all transcripts moderately induced/repressed at 1.5 or 24 hours after IR treatment (fold change > 2 or < -2 and adjusted p-value < 0.05 in response to HZE or gamma radiation, for WT or *atm-1* plants), we display expression profiles across the full IR time series as well as across circadian and diurnal time series. Circadian and diurnal profiles were scaled- separately, and for each transcript- so that the minimum fold change would be 0. Each column indicates a particular experimental condition (combination of stress, time point, and spatial region). The profiles are clustered (row clustering) only in terms of the expression values at 1.5 or 24 hours after IR treatment. Intermediate time points of 3, 6, or 12 hours after IR (as well as the circadian and diurnal time series) were added after clustering was performed. The gray vertical bars indicate a missing time point at 3 hours for the *atm-1* mutant. Horizontal gray bars indicate the selection of transcripts in each cluster A-E. Labels represent significant enrichment for selected GO terms (see text).

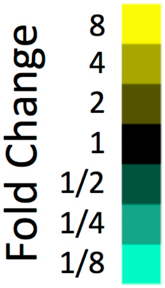

Supplement: Supplementary file 6 [file Presentation6.PDF]
