# Supplementary material for: High atomic weight, high-energy radiation (HZE) induces transcriptional responses shared with conventional stresses in addition to a core “DSB” response specific to clastogenic treatments
Source: Front Plant Sci. 2014 Aug 1;5:364. doi: 10.3389/fpls.2014.00364 (PMC4117989; doi:10.3389/fpls.2014.00364)
Supplement: Supplementary file 8 [file DataSheet2.DOCX]

**Table S2. Human Homologs.**

| query ids | subject ids | % identity | % positives | evalue | bit score | Arabidopsis annotation | Human annotation |
| --- | --- | --- | --- | --- | --- | --- | --- |
| AT5G20850 | gi\|19924133\|ref\|NP_002866.2 | 71.3 | 87.35 | 6.0E-177 | 499 | ATRAD51_RAD51__RAS associated with diabetes protein 51 | DNA repair protein RAD51 homolog 1 isoform 1... |
| AT4G02390 | gi\|110825963\|ref\|NP_001036083.1 | 47.79 | 65.46 | 9.0E-147 | 442 | APP_ATPARP1_PARP1_poly(ADP-ribose) polymerase | poly [ADP-ribose] polymerase 2 isoform 2 ... |
| AT5G20930 | gi\|530411410\|ref\|XP_005257030.1 | 51.12 | 65.18 | 4.0E-132 | 409 | TSL__Protein kinase superfamily protein | PREDICTED: tousled-like kinase 2 isoform ... |
| AT2G23150 | gi\|109255241\|ref\|NP_000569.3 | 51.14 | 67.27 | 3.0E-135 | 407 | ATNRAMP3_NRAMP3__natural resistance-associated macrophage protein 3 | natural resistance-associated macrophage pro... |
| AT1G31280 | gi\|29171734\|ref\|NP_036286.2 | 31.96 | 52.13 | 1.0E-124 | 405 | AGO2__Argonaute family protein | protein argonaute-2 isoform 1 [Homo sapiens] |
| AT3G21280 | gi\|4827050\|ref\|NP_005142.1 | 42.06 | 60.32 | 9.0E-132 | 397 | UBP7__ubiquitin-specific protease 7 | ubiquitin carboxyl-terminal hydrolase 14 iso... |
| AT2G31320 | gi\|110825961\|ref\|NP_005475.2 | 39.66 | 61.1 | 1.0E-112 | 364 | ATPARP2_PARP2__poly(ADP-ribose) polymerase 2 | poly [ADP-ribose] polymerase 2 isoform 1 [Ho... |
| AT4G35740 | gi\|530413170\|ref\|XP_005257880.1 | 43.39 | 61.25 | 3.0E-114 | 360 | ATRECQ3_RecQl3__DEAD/DEAH box RNA helicase family protein | PREDICTED: ATP-dependent DNA helicase Q5 ... |
| AT2G06510 | gi\|4506583\|ref\|NP_002936.1 | 35.12 | 55.35 | 2.0E-107 | 341 | ATRPA1A_ATRPA70A_RPA1A_RPA70A__replication protein A 1A | replication protein A 70 kDa DNA-binding sub... |
| AT2G18760 | gi\|58331268\|ref\|NP_060139.2 | 32.14 | 52.82 | 4.0E-92 | 325 | CHR8__chromatin remodeling 8 | DNA excision repair protein ERCC-6-like [Hom... |
| AT5G45400 | gi\|4506583\|ref\|NP_002936.1 | 39.73 | 59.59 | 3.0E-98 | 323 | ATRPA70C_RPA70C__Replication factor-A protein 1-related | replication protein A 70 kDa DNA-binding sub... |
| AT5G66140 | gi\|68303563\|ref\|NP_001020267.1 | 69.91 | 84.26 | 4.0E-108 | 317 | PAD2__proteasome alpha subunit D2 | proteasome subunit alpha type-7-like isof... |
| AT4G19130 | gi\|4506583\|ref\|NP_002936.1 | 38.93 | 60.63 | 8.0E-94 | 310 | Replication factor-A protein 1-related | replication protein A 70 kDa DNA-binding sub... |
| AT5G15540 | gi\|530378766\|ref\|XP_005248340.1 | 26.7 | 46.25 | 1.0E-84 | 310 | ATSCC2_EMB2773_SCC2__PHD finger family protein | PREDICTED: nipped-B-like protein isoform ... |
| AT2G21300 | gi\|530377087\|ref\|XP_005262756.1 | 39.95 | 58.39 | 1.0E-75 | 274 | ATP binding microtubule motor family protein | PREDICTED: centromere-associated protein ... |
| AT5G08380 | gi\|4504009\|ref\|NP_000160.1 | 42.06 | 57.35 | 1.0E-80 | 259 | AGAL1_AtAGAL1__alpha-galactosidase 1 | alpha-galactosidase A precursor [Homo sapiens] |
| AT5G49990 | gi\|40316845\|ref\|NP_005107.4 | 34.39 | 52.52 | 8.0E-75 | 253 | Xanthine/uracil permease family protein | solute carrier family 23 member 2 [Homo sapi... |
| AT2G41630 | gi\|4504193\|ref\|NP_001505.1 | 46.9 | 62.76 | 2.0E-78 | 246 | TFIIB__transcription factor IIB | transcription initiation factor IIB [Homo sa... |
| AT2G30360 | gi\|530403740\|ref\|XP_005267700.1 | 44.49 | 65.78 | 7.0E-73 | 245 | CIPK11_PKS5_SIP4_SNRK3.22__SOS3-interacting protein 4 | PREDICTED: MAP/microtubule affinity-regul... |
| AT2G18600 | gi\|4507791\|ref\|NP_003960.1 | 62.16 | 78.92 | 4.0E-80 | 241 | Ubiquitin-conjugating enzyme family protein | NEDD8-conjugating enzyme Ubc12 [Homo sapiens] |
| AT3G13235 | gi\|62955833\|ref\|NP_115717.3 | 36.18 | 57.83 | 7.0E-72 | 235 | DDI1__ubiquitin family protein | protein DDI1 homolog 2 [Homo sapiens] |
| AT4G28950 | gi\|9845511\|ref\|NP_008839.2 | 63.79 | 75.29 | 3.0E-76 | 232 | ARAC7_ATRAC7_ATROP9_RAC7_ROP9__RHO-related protein from plants 9 | ras-related C3 botulinum toxin substrate 1 i... |
| AT3G60070 | gi\|111378391\|ref\|NP_778148.2 | 32.69 | 53.61 | 9.0E-61 | 209 | Major facilitator superfamily protein | major facilitator superfamily domain-contain... |
| AT5G57220 | gi\|4503199\|ref\|NP_000490.1 | 28.51 | 47.33 | 9.0E-60 | 208 | CYP81F2__cytochrome P450, family 81, subfamily F, polypeptide 2 | cytochrome P450 1A1 [Homo sapiens] >ref |
| AT1G09180 | gi\|7705827\|ref\|NP_057187.1 | 60 | 75.38 | 2.0E-66 | 207 | ATSAR1_ATSARA1A_SARA1A__secretion-associated RAS super family 1 | GTP-binding protein SAR1b [Homo sapiens] >re... |
| AT4G37490 | gi\|161377470\|ref\|NP_001104516.1 | 38.65 | 57.8 | 2.0E-53 | 188 | CYC1_CYCB1_CYCB1;1__CYCLIN B1;1 | cyclin-A1 isoform c [Homo sapiens] >ref |
| AT3G25250 | gi\|530421221\|ref\|XP_005274631.1 | 34.15 | 50.27 | 8.0E-52 | 187 | AGC2_AGC2-1_AtOXI1_OXI1__AGC (cAMP-dependent | PREDICTED: ribosomal protein S6 kinase al... |
| AT5G49110 | gi\|530407220\|ref\|XP_005255009.1 | 27.59 | 49.43 | 4.0E-47 | 187 | unknown protein; | PREDICTED: Fanconi anemia group I protein... |
| AT3G57550 | gi\|4504221\|ref\|NP_000849.1 | 48.62 | 68.51 | 1.0E-55 | 186 | AGK2_GK-2__guanylate kinase | guanylate kinase isoform b [Homo sapiens] >r... |
| AT2G47680 | gi\|67782362\|ref\|NP_061903.2 | 29.53 | 46.49 | 2.0E-47 | 186 | zinc finger (CCCH type) helicase family protein | ATP-dependent RNA helicase DHX29 [Homo sapiens] |
| AT3G24515 | gi\|7661808\|ref\|NP_054895.1 | 53.16 | 71.52 | 9.0E-54 | 181 | UBC37__ubiquitin-conjugating enzyme 37 | ubiquitin-conjugating enzyme E2 T [Homo sapi... |
| AT1G07650 | gi\|223671883\|ref\|NP_001138728.1 | 37.26 | 55.41 | 2.0E-46 | 173 | Leucine-rich repeat transmembrane protein kinase | interleukin-1 receptor-associated kinase ... |
| AT4G29170 | gi\|14149769\|ref\|NP_115493.1 | 43.75 | 64.42 | 4.0E-51 | 169 | ATMND1__Mnd1 family protein | meiotic nuclear division protein 1 homolog i... |
| AT2G31870 | gi\|530394561\|ref\|XP_005270311.1 | 40.65 | 55.69 | 4.0E-44 | 169 | PARG1_TEJ__Poly (ADP-ribose) glycohydrolase (PARG) | PREDICTED: poly(ADP-ribose) glycohydrolas... |
| AT1G79820 | gi\|5902090\|ref\|NP_008862.1 | 30.29 | 48.78 | 3.0E-44 | 166 | SGB1__Major facilitator superfamily protein | solute carrier family 2, facilitated glucose... |
| AT1G07570 | gi\|223671883\|ref\|NP_001138728.1 | 36.31 | 57.23 | 4.0E-44 | 160 | APK1_APK1A__Protein kinase superfamily protein | interleukin-1 receptor-associated kinase ... |
| AT3G13380 | gi\|223671883\|ref\|NP_001138728.1 | 35.88 | 53.16 | 6.0E-42 | 160 | BRL3__BRI1-like 3 | interleukin-1 receptor-associated kinase ... |
| AT4G22790 | gi\|153792564\|ref\|NP_001093116.1 | 30.11 | 50.11 | 1.0E-38 | 149 | MATE efflux family protein | multidrug and toxin extrusion protein 2 i... |
| AT1G09870 | gi\|19923761\|ref\|NP_004888.2 | 26.59 | 45.37 | 3.0E-37 | 145 | histidine acid phosphatase family protein | multiple inositol polyphosphate phosphatase ... |
| AT5G66130 | gi\|19718786\|ref\|NP_579917.1 | 28.6 | 46.02 | 4.0E-35 | 142 | ATRAD17_RAD17__RADIATION SENSITIVE 17 | cell cycle checkpoint protein RAD17 isoform ... |
| AT3G07800 | gi\|164698438\|ref\|NP_003249.3 | 45.6 | 62.09 | 4.0E-39 | 139 | Thymidine kinase | thymidine kinase, cytosolic [Homo sapiens] |
| AT5G23910 | gi\|372266146\|ref\|NP_001243198.1 | 30.9 | 52.08 | 5.0E-33 | 135 | ATP binding microtubule motor family protein | kinesin-like protein KIF22 isoform 2 [Hom... |
| AT3G53280 | gi\|530377126\|ref\|XP_005262775.1 | 26.91 | 46.17 | 2.0E-33 | 134 | CYP71B5__cytochrome p450 71b5 | PREDICTED: cytochrome P450 2U1 isoform X2... |
| AT1G59660 | gi\|56549643\|ref\|NP_005378.4 | 40.23 | 58.62 | 6.0E-31 | 132 | Nucleoporin autopeptidase | nuclear pore complex protein Nup98-Nup96 iso... |
| AT1G01220 | gi\|530423659\|ref\|XP_005255890.1 | 31.94 | 50.45 | 2.0E-30 | 131 | AtFKGP_FKGP__L-fucokinase/GDP-L-fucose pyrophosphorylase | PREDICTED: L-fucose kinase isoform X2 [Ho... |
| AT2G03870 | gi\|7706423\|ref\|NP_057283.1 | 55.32 | 78.72 | 2.0E-35 | 120 | EMB2816__Small nuclear ribonucleoprotein family protein | U6 snRNA-associated Sm-like protein LSm7 [Ho... |
| AT3G09020 | gi\|8392830\|ref\|NP_059132.1 | 30.43 | 49.28 | 2.0E-29 | 119 | alpha 1,4-glycosyltransferase family protein | lactosylceramide 4-alpha-galactosyltransfera... |
| AT3G08620 | gi\|530384229\|ref\|XP_005267282.1 | 47.76 | 72.39 | 8.0E-30 | 117 | RNA-binding KH domain-containing protein | PREDICTED: protein quaking isoform X2 [Ho... |
| AT3G17250 | gi\|75813618\|ref\|NP_001028728.1 | 29.21 | 48.45 | 4.0E-29 | 117 | Protein phosphatase 2C family protein | protein phosphatase 1B isoform 4 [Homo sa... |
| AT1G13330 | gi\|7706577\|ref\|NP_057640.1 | 37 | 57 | 2.0E-26 | 104 | AHP2__Arabidopsis Hop2 homolog | homologous-pairing protein 2 homolog isoform... |
| AT4G21070 | gi\|543583789\|ref\|NP_001269474.1 | 37.5 | 51.14 | 2.0E-22 | 101 | ATBRCA1_BRCA1__breast cancer susceptibility1 | BRCA1-associated RING domain protein 1 is... |
| AT3G18940 | gi\|22726189\|ref\|NP_064617.2 | 28.68 | 47.79 | 5.0E-24 | 100 | clast3-related | proteasome assembly chaperone 2 isoform 1 [H... |
| AT4G22960 | gi\|253795490\|ref\|NP_001156731.1 | 38.46 | 55.77 | 1.0E-22 | 100 | Protein of unknown function (DUF544) | protein FAM63A isoform 4 [Homo sapiens] |
| AT1G68200 | gi\|530404540\|ref\|XP_005268083.1 | 55.13 | 64.1 | 2.0E-23 | 99.8 | Zinc finger C-x8-C-x5-C-x3-H type family protein | PREDICTED: ZFP36 ring finger protein-like... |
| AT1G69420 | gi\|530435177\|ref\|XP_005276313.1 | 35.53 | 55.26 | 3.0E-20 | 92.8 | DHHC-type zinc finger family protein | PREDICTED: probable palmitoyltransferase ... |
| AT4G35030 | gi\|68800343\|ref\|NP_001020413.1 | 36.36 | 53.9 | 9.0E-26 | 91.3 | Protein kinase superfamily protein | interleukin-1 receptor-associated kinase ... |
| AT5G40840 | gi\|5453994\|ref\|NP_006256.1 | 37.69 | 57.69 | 2.0E-18 | 90.9 | AtRAD21.1_SYN2__Rad21/Rec8-like family protein | double-strand-break repair protein rad21 hom... |
| AT1G22510 | gi\|237858650\|ref\|NP_001153695.1 | 41.12 | 57.94 | 9.0E-21 | 89 | RING/U-box protein with domain of unknown function (DUF 1232) | E3 ubiquitin-protein ligase RNF170 isofor... |
| AT5G60250 | gi\|187761373\|ref\|NP_005735.2 | 29.07 | 43.17 | 4.0E-17 | 86.7 | zinc finger (C3HC4-type RING finger) family protein | E3 ubiquitin-protein ligase ARIH1 [Homo sapi... |
| AT5G51740 | gi\|530361710\|ref\|XP_005270482.1 | 33.33 | 51.15 | 1.0E-17 | 84 | Peptidase family M48 family protein | PREDICTED: metalloendopeptidase OMA1, mit... |
| AT3G42860 | gi\|187608726\|ref\|NP_001120664.1 | 31.58 | 47.37 | 9.0E-17 | 79.3 | zinc knuckle (CCHC-type) family protein | cellular nucleic acid-binding protein iso... |
| AT3G27320 | gi\|206597554\|ref\|NP_997248.2 | 24.23 | 37.12 | 1.0E-14 | 77 | alpha/beta-Hydrolases superfamily protein | arylacetamide deacetylase-like 2 precursor [... |
| AT4G19670 | gi\|556562062\|ref\|NP_001273327.1 | 26.17 | 43.93 | 4.0E-14 | 76.3 | RING/U-box superfamily protein | probable E3 ubiquitin-protein ligase RNF2... |
| AT5G58070 | gi\|4502163\|ref\|NP_001638.1 | 33.74 | 48.47 | 2.0E-15 | 72.4 | ATTIL_TIL__temperature-induced lipocalin | apolipoprotein D precursor [Homo sapiens] |
| AT5G10650 | gi\|377520133\|ref\|NP_001243687.1 | 35.29 | 50 | 1.0E-13 | 70.9 | RING/U-box superfamily protein | RING finger protein 165 isoform 2 [Homo s... |
| AT2G20320 | gi\|55749789\|ref\|NP_079096.2 | 31.25 | 50 | 3.0E-11 | 68.6 | DENN (AEX-3) domain-containing protein | DENN domain-containing protein 1A isoform 2 ... |
| AT2G46610 | gi\|311771542\|ref\|NP_001185773.1 | 26.71 | 46.58 | 1.0E-13 | 68.2 | At-RS31a_RS31a__RNA-binding (RRM/RBD/RNP motifs) family protein | RNA-binding protein 4 isoform 3 [Homo sap... |
| AT3G15180 | gi\|4826952\|ref\|NP_005038.1 | 21.73 | 39.87 | 3.0E-11 | 67 | ARM repeat superfamily protein | 26S proteasome non-ATPase regulatory subunit... |
| AT5G15380 | gi\|28559065\|ref\|NP_787046.1 | 34.93 | 47.95 | 8.0E-11 | 66.6 | DRM1__domains rearranged methylase 1 | DNA (cytosine-5)-methyltransferase 3B isofor... |
| AT5G49480 | gi\|4502549\|ref\|NP_001734.1 | 29.41 | 52.94 | 2.0E-13 | 65.9 | ATCP1_CP1__Ca2+-binding protein 1 | calmodulin [Homo sapiens] >ref |
| AT1G07350 | gi\|4502847\|ref\|NP_001271.1 | 37.33 | 65.33 | 2.0E-10 | 60.8 | SR45a__RNA-binding (RRM/RBD/RNP motifs) family protein | cold-inducible RNA-binding protein [Homo sap... |
| AT1G09815 | gi\|258547140\|ref\|NP_066996.3 | 56.1 | 75.61 | 5.0E-11 | 57.8 | POLD4__polymerase delta 4 | DNA polymerase delta subunit 4 isoform 1 [Ho... |

**Table S2.** 72 BLAST alignments with e-values less than 1e-6 for the 160 shared IR-induced transcripts at 1.5 hours after HZE or gamma. Known genes involved in DSB repair, DNA replication, DNA methylation and cell cycle control are highlighted.
